# Functional and Genomic Characterization of *Ligilactobacillus salivarius* TUCO-L2 Isolated From *Lama glama* Milk: A Promising Immunobiotic Strain to Combat Infections

**DOI:** 10.3389/fmicb.2020.608752

**Published:** 2020-12-08

**Authors:** Sandra Quilodrán-Vega, Leonardo Albarracin, Flavia Mansilla, Lorena Arce, Binghui Zhou, Md Aminul Islam, Mikado Tomokiyo, Imad Al Kassaa, Yoshihito Suda, Haruki Kitazawa, Julio Villena

**Affiliations:** ^1^Laboratory of Food Microbiology, Faculty of Veterinary Sciences, University of Concepción, Chillán, Chile; ^2^Laboratory of Immunobiotechnology, Reference Centre for Lactobacilli (CERELA-CONICET), Tucumán, Argentina; ^3^Food and Feed Immunology Group, Laboratory of Animal Products Chemistry, Graduate School of Agricultural Science, Tohoku University, Sendai, Japan; ^4^Laboratory of Computing Science, Faculty of Exact Sciences and Technology, Tucuman University, Tucuman, Argentina; ^5^Infection Biology Laboratory, Instituto Superior de Investigaciones Biológicas (INSIBIO-CONICET), Tucumán, Argentina; ^6^Livestock Immunology Unit, International Education and Research Center for Food and Agricultural Immunology (CFAI), Graduate School of Agricultural Science, Tohoku University, Sendai, Japan; ^7^Department of Medicine, Faculty of Veterinary Science, Bangladesh Agricultural University, Mymensingh, Bangladesh; ^8^Faculty of Public Health, Lebanese University, Hadath, Lebanon; ^9^Department of Food, Agriculture and Environment, Miyagi University, Sendai, Japan

**Keywords:** *Ligilactobacillus salivarius* TUCO-L2, *Lama glama* milk, bacterial infection, probiotic, immunomodulation, intestinal epithelia cell

## Abstract

Potential probiotic or immunobiotic effects of lactic acid bacteria (LAB) isolated from the milk of the South American camelid llama (*Lama glama*) have not been reported in published studies. The aim of the present work was to isolate beneficial LAB from llama milk that can be used as potential probiotics active against bacterial pathogens. LAB strains were isolated from llama milk samples. *In vitro* functional characterization of the strains was performed by evaluating the resistance against gastrointestinal conditions and inhibition of the pathogen growth. Additionally, the adhesive and immunomodulatory properties of the strains were assessed. The functional studies were complemented with a comparative genomic evaluation and *in vivo* studies in mice. *Ligilactobacillus salivarius* TUCO-L2 showed enhanced probiotic/immunobiotic potential compared to that of other tested strains. The TUCO-L2 strain was resistant to pH and high bile salt concentrations and demonstrated antimicrobial activity against Gram-negative intestinal pathogens and adhesion to mucins and epithelial cells. *L. salivarius* TUCO-L2 modulated the innate immune response triggered by Toll-like receptor (TLR)-4 activation in intestinal epithelial cells. This effect involved differential regulation of the expression of inflammatory cytokines and chemokines mediated by the modulation of the negative regulators of the TLR signaling pathway. Moreover, the TUCO-L2 strain enhanced the resistance of mice to *Salmonella* infection. This is the first report on the isolation and characterization of a potential probiotic/immunobiotic strain from llama milk. The *in vitro, in vivo*, and *in silico* investigation performed in this study reveals several research directions that are needed to characterize the TUCO-L2 strain in detail to position this strain as a probiotic or immunobiotic that can be used against infections in humans or animals, including llama.

## Introduction

The Camelidae family comprises the dromedary camel or camel of the plains (*Camelus dromedarius*), Bactrian camel or camel of the mountains (*Camelus bactrianus*), and four species of South American camelids, including guanaco (*Lama guanicoe*), vicuña (*Vicugna vicugna*), alpaca (*Lama pacos*), and llama (*Lama glama*) (Fukuda, 2013; Zarrin et al., 2020). The milk of camels has been a part of the daily diet of nomadic people living in the steppe and arid areas of central Asia for centuries because of its good nutritional properties (Shabo et al., 2005). The beneficial effects of the camel milk are attributed to its nutritional composition, physicochemical characteristics, and rich microbial population (Dong et al., 2012; Jans et al., 2012). Therefore, in the past decade, considerable efforts were aimed at characterizing the microbial populations of the camel milk to isolate the beneficial strains.

Several studies described the complex population of lactic acid bacteria (LAB) in the camel milk. Earlier studies reported the presence of *Lactobacillus* spp., *Lactococcus* spp., *Streptococcus* spp., and *Enterococcus* spp. in the milk of camels (Khedid et al., 2009). Moreover, a study reported that the most abundant cultivable species of Gram-positive cocci included *Enterococcus faecium* and *Lactococcus lactis*; the most abundant lactobacilli species included *Lactobacillus helveticus, Lacticaseibacillus casei* (basonym: *Lactobacillus casei* (Zheng et al., 2020), and *Lactiplantibacillus plantarum* (basonym: *Lactobacillus plantarum*). Subsequent studies confirmed the dominance of enterococci and lactococci in the cultivable bacteria from camel milk but detected certain differences in the predominant species of lactobacilli (Elbanna et al., 2018; Rahmeh et al., 2019; Zhao et al., 2020) that included *Lacticaseibacillus paracasei* (basonym: *Lactobacillus paracasei*), *Lacticaseibacillus rhamnosus* (basonym: *Lactobacillus rhamnosus*), and *Limosilactobacillus reuteri* (basonym: *Lactobacillus reuteri*). In addition to the characterization of microbial populations, certain strains with beneficial effects have been isolated from the camel milk. A strain isolated from fermented camel milk, *L. casei* TN-2, produces a bacteriocin that inhibits *Escherichia coli* and *Staphylococcus aureus* (Lü et al., 2014). The *E. faecium* LCW44 strain isolated from raw camel milk demonstrated a pronounced inhibitory effect against *Listeria* spp. and *S. aureus* (Vimont et al., 2017). The *Lactobacillus acidophilus* AA105 strain from camel milk is a potent inhibitor of *Salmonella paratyphi, Shigella* spp., and pathogenic *E. coli* (Abo-Amer, 2013), and *L. brevis* CM22 demonstrated inhibitory effects against *Listeria* spp. (Rahmeh et al., 2019). Interestingly, a recent report of Elbanna et al. (2018) showed that *L. paracasei* Pro4 and *L. rhamnosus* Pro7 isolated from fermented camel milk modulate the intestinal immunity after oral administration in mice by improving the expression of Toll-like receptor (TLR)-2, interferon (IFN)-γ, and secretory immunoglobulin A (IgA) indicating that LAB isolated from the milk of camelids can be beneficial modulators of the immune system.

In South America, the guanaco and vicuña camelids are wild animals, and alpaca and llama are domesticated species (Zarrin et al., 2020). It is estimated that there are approximately 5 million llamas in South America (FAOSTAT, 2019). Andean native communities mainly use llamas as pack animals, for meat production, and, to a lesser extent, for production of fiber and milk (Pérez et al., 2000; Larico et al., 2018). Llama meat has been characterized and was shown to be rich in iron and zinc (Polidori et al., 2007) and contain low fat and cholesterol compared with those in the meat of other livestock species (Cristofanelli et al., 2004). On the other hand, although milk yield in llamas is very low compared with that in dairy cows, llama milk has higher levels of protein, fat, and lactose than those in the cow milk; these properties suggest that llama milk can be used to develop new dairy products (Larico et al., 2018; Zarrin et al., 2020). These characteristics of llama milk suggest that it can be used as an alternative to cow milk; however, there are no in-depth studies on the biotechnological and functional potential of llama milk. Studies on the nutritional properties and impact on human health of llama milk are required to position it as a safe and healthy food that can expand globally in the future (Pauciullo and Erhardt, 2015). The composition of the microbial populations and characterization of derived strains are the important factors that should be analyzed in llama milk. These strains can be used: (a) to develop safe and varied dairy products, (b) to improve the health and productivity of llamas, and (c) to provide beneficial modulation of human health.

To the best of our knowledge, there are no published reports on the potential probiotic effects of bacteria isolated from the milk of llama. The aim this study was to isolate beneficial LAB from the llama milk and use them as potential probiotics against bacterial pathogens. LAB strains were isolated from the llama milk samples, and their functional properties were characterized *in vitro* based on their ability to resist gastrointestinal conditions, adhere to intestinal mucins and cells, inhibit the growth of the pathogens, and differentially modulate the TLR4-mediated innate immune responses in intestinal epithelial cells. These functional studies were complemented with a comparative genomic evaluation and *in vivo* studies in mice.

## Materials and Methods

### Isolation of LAB From Llama Milk

Five llama milk samples were collected aseptically by trained personnel of the Laboratory of Food Microbiology (Faculty of Veterinary Sciences, University of Concepción) in the Bio-Bio region of Chile and transported in cold containers. Resistance to pH 3 was used as a selection criterion to isolate LAB from llama milk as described previously (Quilodrán-Vega et al., 2016). Briefly, 150 μl of each milk sample was placed in MRS broth and incubated at 37°C at 5% CO_2_ for 12 h. Subsequently, 100 μl of the bacteria grown in MRS were transferred to new MRS broth adjusted to pH 3 with 5N HCl. The new MRS broth cultures were incubated for additional 3 h under the same conditions. The cultures were transferred to MRS agar, and grown colonies were subjected to the Gram staining and catalase test. Colonies of Gram-positive and catalase-negative microorganisms resistant to pH 3 were selected for subsequent studies.

### Identification of Isolated Strains

The isolates confirmed as Gram-positive and catalase-negative bacilli were biochemically identified using the API 50 CH test as described elsewhere (Brolazo et al., 2011). Strains of interest were further identified using species-specific primers as described previously (Quilodrán-Vega et al., 2016). Briefly, genomic DNA extraction was performed using a ZR fungal/bacterial DNA miniprep kit (Zymo Research, CA, U.S.A., catalog no. D6005). Positive strains of the *Lactobacillus* genus were subjected to PCR analysis using the genera-specific primers as described previously (Quilodrán-Vega et al., 2016). *Salmonella* Typhimurium and *L. rhamnosus* CRL1505 DNA samples were used as negative and positive controls, respectively. A single bacterial strain, *Ligilactobacillus salivarius* TUCO-L2 (basonym: *Lactobacillus salivarius* TUCO-L2), showing potential probiotic properties was further identified by 16S RNA and complete genome sequencing (DDBJ/ENA/GenBank under the accession number SOPE01000000) (Albarracin et al., 2020b).

### Screening of LAB

Screening for the selection of potential probiotic strains was based on resistance to NaCl and Oxgall (bile salts). LAB strains were cultivated in MRS broth containing 3, 6.5, or 9% w/v NaCl and in MRS broth containing 0.3, 0.6, 2, 3, or 5% w/v Oxgall as described previously (Quilodrán-Vega et al., 2016). Bacteria were incubated for 5 days at 37°C at 5% CO_2_, and 100 μl of each tube was streaked on MRS agar to test viability.

### Inhibition of Bacterial Pathogens

Various intestinal pathogens were used to evaluate the antagonistic effect of lactobacilli isolated from llama milk. The human intestinal pathogens *Salmonella enterica* ATCC 13076 and *Escherichia coli* ATCC 25922 were obtained from Oxoid (Argentina). The porcine pathogens enterotoxigenic *E. coli* TUCO-I5, enterohemorragic *E. coli* TUCO-I6, and *Salmonella* Typhimurium TUCO-I7 were obtained from the pathogen culture collection of the Laboratory of Food Microbiology (Faculty of Veterinary Sciences, University of Concepción). *Escherichia coli* and *Salmonella* strains were grown in BHI broth under the standard conditions (Quilodrán-Vega et al., 2016).

The study of inhibition of bacterial pathogens was performed as described previously (Quilodrán-Vega et al., 2016). Briefly, a 10 μl loop of a 2-day culture on MRS broth of the LAB strains was streaked on a line on MRS agar and incubated at 37°C at 5% CO_2_ for 24 h. After incubation, a 10 μl loop of overnight cultures from the pathogen strains was streaked perpendicular to the lactobacilli strains lines. The plates were incubated at 37°C for 48 h under aerobic conditions. The inhibition zones were measured in cm.

### Autoaggregation and Coaggregation

LAB were cultivated in MRS broth for 48 h at 37°C, and the cells were harvested by centrifugation for 10 min at 10,000 × g. The pellet was washed twice with Butterfield's buffer (pH 7.2) and resuspended in 4 ml of new MRS broth. The bacterial concentration in the new MRS broth was adjusted to 0.5 MacFarland in a DensiCHEK™ Plus instrument. The tubes were incubated for 6 or 24 h at 37°C. Then, samples were withdrawn from the top of the suspensions. Autoaggregation was calculated using the equation: autoaggregation (%) = ((A0 – At)/A0) × 100, where A0 indicates the absorbance at time 0 h and At indicates the absorbance at 6 or 24 h.

LAB suspensions for the coaggregation assay were prepared as described above. Additionally, the suspensions of pathogenic bacteria grown in BHI were prepared in a similar manner and adjusted to 0.5 MacFarland. Each LAB suspension (1 ml) was mixed with the same volume of each pathogen suspension. The mixtures were vortexed and left for gravity sedimentation. Tubes containing 2 ml of each pathogenic bacteria suspension without LAB were used as the controls. The concentration in MacFarland of the suspensions was determined after incubation at 37°C for 6 or 24 h. Coaggregation was calculated using the equation: coaggregation (%) = [(Alab + Apat)/2–A(lab+pat)]/[(Alab + Apat)/2], where lab and pat indicate the absorbance of the LAB and pathogen strains, respectively; and (lab+pat) indicates the absorbance of the mixtures.

### Scanning Electron Microscopy (SEM) Analysis

The strain with good probiotic characteristics (*L. salivarius* TUCO-L2) was washed once and diluted two-fold with PBS. The bacterial suspension was dropped on a polycarbonate membrane (ADVANTEC) and filtered by vacuum filtration (Millipore). The membrane with lactobacilli on the surface was immersed in 2% (v/v) glutaraldehyde solution. After 1 h, the membrane was dehydrated by sequential immersion in 50, 60, 70, 80, 90, and 99% ethanol for 20 min at each step. The membrane was immersed in t-butyl alcohol and lyophilized. SEM was performed in an electron microscopy facility (CIME-CONICET-UNT, Tucuman, Argentina).

### PIE Cells

The PIE cell line was originally established at Tohoku University from intestinal epithelium of an unsuckled neonatal pig as described previously (Moue et al., 2008). After 3 days of culture, PIE cells form a monolayer with cobblestone- and epithelial-like morphology and close contacts between the cells. PIE cells are strongly positive for cytokeratin K8.13, a marker of porcine intestinal epithelial cells (Moue et al., 2008). PIE cells grow rapidly and are well-adapted to culture conditions even without transformation or immortalization. However, the proliferation of PIE cells diminishes after 50 passages in culture. Therefore, in the experiments, PIE cells only from passage 20 to 40 were used (Albarracin et al., 2017). DMEM medium supplemented with 10% fetal calf serum (FCS), penicillin (100 mg/ml), and streptomycin (100 U/ml) was used for maintenance of PIE cells. The cells (3.0 × 10^4^ per well) were grown in 12 well type I collagen-coated plates at 37°C in a humidified atmosphere of 5% CO_2_. DMEM containing *L. salivarius* TUCO-L2 (5 × 10^7^ cells/ml; 1 ml) was added to PIE cell monolayers. The cells were incubated for 48 h at 37°C at 5% CO_2_. PIE cells were washed with fresh medium to remove lactobacilli and stimulated with heat-stable pathogen associated molecular patterns (PAMPs) from enterotoxigenic *Escherichia coli* (ETEC) for 12 h to induce the activation of TLR4 as described previously (Shimazu et al., 2012; Garcia-Castillo et al., 2019). PIE cells not treated with lactobacilli and challenged with ETEC PAMPs were used as a control. PIE cells without any stimulation were used for comparison and are designated as a basal control.

The expression levels of interleukin (*IL*)-*1*β*, IL-6*, chemokine C-C motif ligand 8 (*CCL8* or *MCP2*), C-X-C motif chemokine 5 (*CXCL5* or *ENA78*), *CXCL8* or *IL-8, CXCL9* or *MIG, CXCL10* or *IP-9*, and *CXCL11* or *IP-9* were evaluated after TLR4 activation by using RT-qPCR as detailed below. In addition, six negative regulators of TLR signaling were assayed after TLR4 activation: single immunoglobulin IL-1-related receptor (*SIGIRR*), Toll-interacting protein (*Tollip*), interleukin-1 receptor-associated kinase M (*IRAK-M*), ubiquitin-editing enzyme A20 (*A20*), B-cell lymphoma 3-encoded protein (*Bcl-3*), and mitogen-activated protein kinase 1 (*MKP-1*) (Shimazu et al., 2012; Garcia-Castillo et al., 2019).

### RT-qPCR

The expression of immune factors in PIE cells was studied as described previously (Garcia-Castillo et al., 2019; Albarracin et al., 2020a). Briefly, total RNA was extracted with TRIzol reagent (Invitrogen) and its purity and quantity was analyzed by a Nano drop spectrophotometer ND-1000 UV-Vis (NanoDrop Technologies, USA). The RNA (500 ng) was used to synthesize cDNA in a thermal cycler (BIO-RAD, USA) by a Quantitect reverse transcription (RT) kit (Qiagen, Tokyo, Japan) following the manufacturer instructions. The qPCR was performed in a 7,300 real-time PCR system (Applied Biosystems, Warrington, UK) with platinum SYBR green (qPCR supermix containing uracil DNA glycosylase and 5-carboxy-X-rhodamine, Invitrogen). For PCR, 2.5 μl of cDNA was mixed with 7.5 μl of master mix that included SYBR green and forward and reverse primers (1 pmol/μl). The reaction was performed as follows: 50°C for 5 min; 95°C for 5 min; 40 cycles at 95°C for 15 s, 60°C for 30 s, and 72°C for 30 s. β-Actin was used as a housekeeping gene because of its high stability across various porcine tissues (Shimazu et al., 2012; Albarracin et al., 2017). The expression of the housekeeping gene was used to normalize the cDNA levels to account for the differences in total cDNA levels in the samples.

### Biacore Assay for Adhesion to Mucins

Porcine intestinal mucins were used to evaluate the adhesion of *L. salivarius* TUCO-L2. The *L. salivarius* FFIG79 strain highly adhesive to porcine mucin (Masumizu et al., 2019) was used for comparison. Crude mucus was scraped from porcine small intestine. Mucus was digested with 0.5 mg/ml proteinase K (TaKaRa Biotechnology, Shiga, Japan) overnight. After centrifugation (8,500 × g, 4°C, 10 min) and membrane filtration (DISMIC-25, 0.45 μm, Advantec, Tokyo, Japan), the supernatant was purified by gel filtration chromatography over a Toyopearl HW-65F column (90 × 2.6 cm; Tosoh, Tokyo, Japan) using distilled water as the mobile phase. The peptides were detected at 214 nm, and neutral sugar was measured at 490 nm using the phenol-sulfuric acid method. Fractions containing high concentrations of sugars and peptides were collected and concentrated before lyophilization. The purified soluble porcine mucins were used as the ligands for the Biacore analysis.

Biacore experiments were performed using a Biacore 1000 kit (GE Healthcare Bio-Sciences K.K.) at 25°C in HBS-EP buffer. The immobilization of purified porcine mucins on a CM5 sensor chip (GE Healthcare Bio-Sciences K.K.) was performed by the amine coupling reaction following the manufacturer's instructions. Mucins were dissolved at a concentration of 10 mg/ml in 10 mM sodium acetate buffer (pH 4.0) and immobilized using the reaction between N-hydroxysuccinimide (NHS) esters and primary amino groups present in the mucins molecules. The sensor chip was equilibrated in HBS-EP buffer.

Adhesion using Biacore 1,000 is based on the surface plasmon resonance (SPR). After washing and lyophilization, bacterial cells were suspended in HBS-EP buffer (3 mg protein/ml). Bacterial suspension was injected at a flow rate of 3 μl/min for 5 min, and the sensor chip was washed with HBS-EP buffer to remove unbound analyte and regenerated by elution with 1 M guanidine hydrochloride (GHCl) solution at a flow rate of 3 μl/min for 2 min. The resonance units (RU) were determined for 200 s after the end of sample addition. A response of 1 RU represents 1 pg protein/mm^2^ bound to the sensor chip surface at increasing concentrations of the analyte.

### Adhesion to PIE Cells

The adhesion of *L. salivarius* TUCO-L2 to PIE cells was assayed by using the microplate method and fluorescent bacteria. The *L. salivarius* FFIG58 strain highly adhesive to PIE cells (Masumizu et al., 2019) was used for comparison. Cultured lactobacilli were washed with PBS three times (6,000 rpm, 10 min). The pellet was resuspended in 1 ml of PBS, and 1 mM carboxyfluorescein diacetate (CFDA) was added for the fluorescent labeling reaction at 37°C for 1 h. Then, the bacteria were washed with PBS three times (6,000 rpm, 10 min) to remove CFDA on the bacterial surface. Fluorescent bacteria were counted by a hemocytometer.

PIE cells were seeded at 5,000 cells/well in a type I collagen-coated 96 well cell culture plate (Nippi Incorporated, Tokyo) and grown for 3 days. Cultured fluorescent lactobacilli were added to PIE cells at 100 MOI and cocultured for 48 h. After incubation, non-adherent bacteria were washed out with PBS. After lysis with 0.1 N NaOH, fluorescence was evaluated by a 2030 multilabel reader (Perkin Elmer, Madrid, Spain). Wells without PIE cells and labeled with bacteria only were used as the negative controls to determine non-specific fluorescence unrelated to specific adhesion to PIE cells.

### Bioinformatics Analysis

The genome sequence of *L. salivarius* TUCO-L2 was published recently (Albarracin et al., 2020b). The genome sequences of other *L. salivarius* strains from human milk and intestine and pig and chicken intestine were downloaded from the GenBank database (https://www.ncbi.nlm.nih.gov/genome/1207) and used for comparison. Phylogenetic trees were constructed to evaluate the relationships between various microorganisms. The gene sequences were downloaded from the GenBank databases. The MUSCLE aligner (Edgar, 2004) available in the MEGAX (Kumar et al., 2018) software was used to align the gene sequences of all microbes before the construction of the phylogenetic tree according to the neighbor joining (NJ) distance algorithm (Saitou and Nei, 1987; Tamura et al., 2004) embedded in the MEGAX software. The multilocus sequence typing (MLST) analysis was used to construct the trees by the maximum likelihood method based on the sequences of the *parB, rpsB, pheS, nrdB, groEL*, and *ftsQ* genes (Harris et al., 2017; Lee et al., 2017). Heatmaps were constructed using the data plotting tools (Warnes et al., 2017) of R scripts. Pangenome analysis was performed using Roary (v. 3.6.0) (Page et al., 2015) with Prokka annotation (Seemann, 2014). The Venn diagrams were generated using InteractiVenn (Heberle et al., 2015).

### Animals, Feeding Procedures, and Infection

Male 6-week-old Balb/c mice were obtained from CERELA (Tucuman, Argentina). The animals were housed in plastic cages at controlled room temperature (22 ± 2°C, 55 ± 2% humidity) and fed a conventional balanced diet *ad libitum*. Animal welfare was ensured by the investigators and special staff trained in animal care and handling at CERELA. Animal health and behavior were monitored twice a day. This study was carried out in strict accordance with the recommendations in the Guide for the Care and Use of Laboratory Animals of the Guidelines for Animal Experimentation of CERELA. The CERELA Institutional Animal Care and Use Committee prospectively approved this study under the protocol BIOT-CRL-17.

Animals were housed individually during the experiments, and the assays of the parameters were performed in the groups of 5–6 mice. *L. salivarius* TUCO-L2 was administered to mice for 5 consecutive days at a dose of 10^8^ cells/mouse/day in drinking water. The dose was selected according to the preliminary experiments assessing the dose response (unpublished data). The treatment and untreated control groups were fed a conventional balanced diet *ad libitum*. Treated and control mice were challenged with 50 μl of 10^7^ cells/mouse of *S. typhimurium* (20LD50) by oral administration (Quilodrán-Vega et al., 2016). Mice were sacrificed on day 2 after the infection. The liver and spleen of mice of various experimental groups were removed. The organs were homogenized in 0.1% peptone water, appropriately diluted, and plated in MacConkey agar. The plates were incubated at 37°C for 48 h. The results were expressed as the number (log) of CFU/g of organ weight. Bacteremia was monitored in blood samples obtained by cardiac puncture, which were plated on MacConkey agar. The results were expressed as the number (log) of CFU/ml of blood.

The concentrations of the cytokines were determined in the blood and intestinal samples of TUCO-L2-tretated and control mice as described previously (Garcia-Castillo et al., 2019). Briefly, blood samples were obtained by cardiac puncture at the end of each treatment using heparinized tubes. Intestinal fluid samples were obtained by flushing the small intestine with 5 ml of PBS, and the fluid was centrifuged (10,000 × g, 4°C for 10 min) to separate particulate material. The supernatant was stored frozen until use. Tumor necrosis factor α (TNF-α), interferon γ (IFN-γ), IL-1β, IL-6, and IL-10 concentrations in the serum and intestinal fluid were measured with commercially available enzyme-linked immunosorbent assay (ELISA) kits following the manufacturer's recommendations (R&D Systems, MN, USA).

### Statistical Analysis

Statistical analyses were performed using the GLM and REG procedures available in the SAS software (SAS, 1994, https://www.sas.com/en_us/legal/editorial-guidelines.html). Comparison between the mean values was performed using one-way analysis of variance and Fisher least significant difference (LSD) test. For these analyses, *P* < 0.05 were considered significant. For multiple comparisons between mean values, *p* < 0.05 was considered significant and indicated with different superscript letters (a < b < c). The Tukey-Kramer multi comparison was used.

## Results

### Isolation of Potential Probiotic Lactic Acid Bacteria From Llama Milk

A total of 105 different bacterial colonies were isolated from 5 samples of llama milk. The colonies included 41 strains able to grow in MRS broth at pH 3. Evaluation of these 41 LAB strains based on resistance to NaCl and bile salts and on inhibition of *E. coli* ATCC23922 (data not shown) resulted in the selection of four strains for additional experiments: TUCO-L1, TUCO-L2, TUCO-L3, and TUCO-L5. Two bacterial strains were Gram-positive bacilli (TUCO-L2 and TUCO-L5), and TUCO-L1 and TUCO-L3 were Gram-positive cocci. API 50 analysis and molecular biology assays demonstrated that both bacilli strains belong to the *Lactobacillus* genus and cocci belong to the *Enterococcus* genus (data not shown). The four strains had a remarkable ability to grow in the presence of all tested bile salt concentrations (Supplementary Table 1). The TUCO-L1 and TUCO-L2 strains were also resistant to all tested concentrations of NaCl; however, the growth of the TUCO-L3 and TUCO-L5 strains was inhibited by concentrations of NaCl over 6% (w/v) (Supplementary Table 1).

**Figure 1:**
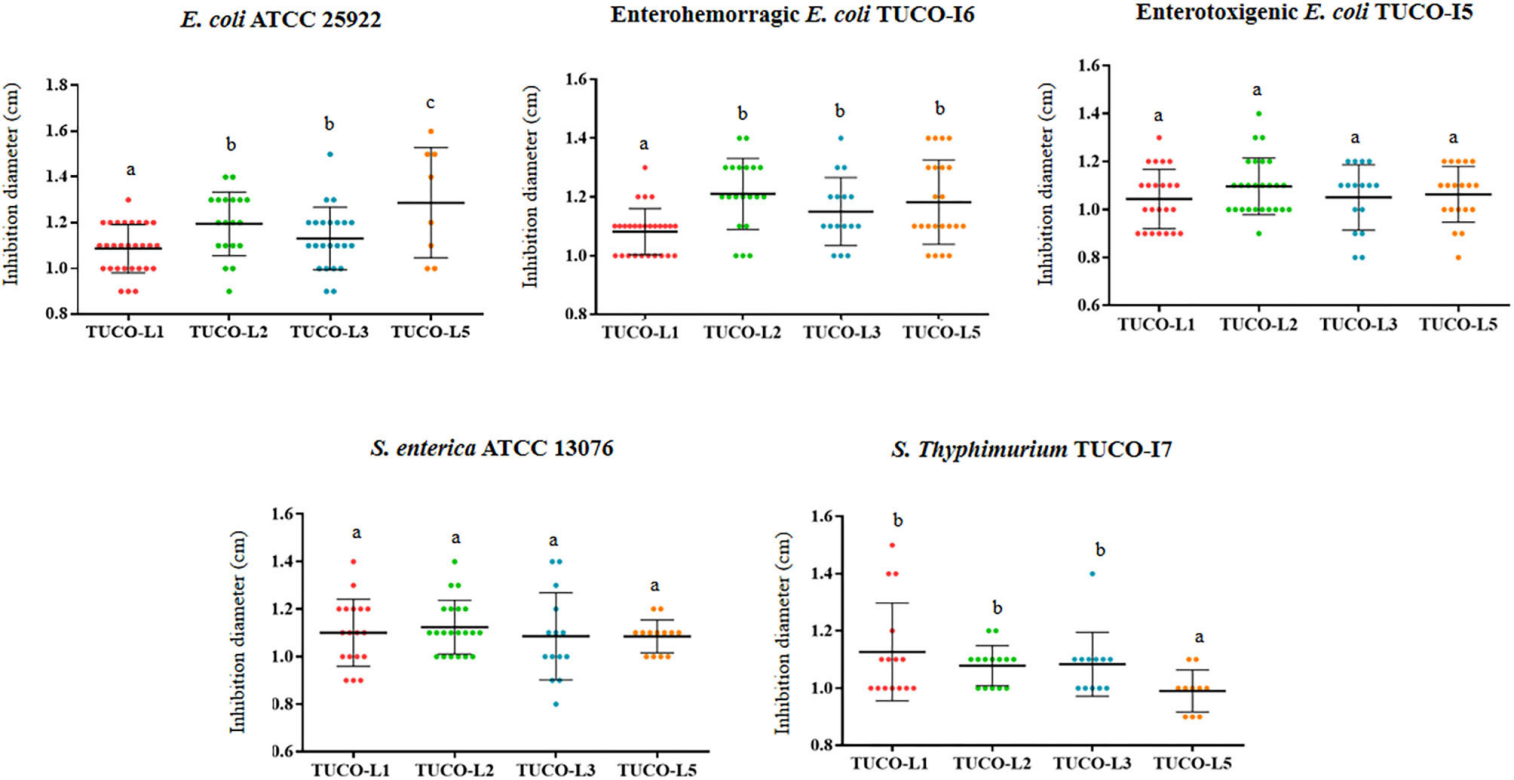
Inhibition of Gram-negative bacterial pathogens by lactic acid bacteria (LAB) isolated from llama (*Lama glama*) milk. The antimicrobial activities of the llama milk strains of lactobacilli (TUCO-L2 and TUCO-L5) and enterococci (TUCO-L1 and TUCO-L3) were evaluated against the human intestinal pathogens *Salmonella enterica* ATCC 13076 and *Escherichia coli* ATCC 25922 and porcine pathogens enterotoxigenic *E. coli* TUCO-I5, enterohemorragic *E. coli* TUCO-I6, and *Salmonella* Typhimurium TUCO-I7. The results represent the data of three independent experiments. Letters indicate significant differences (*P* < 0.05), a < b < c.

The inhibitory potency of the four LAB strains from llama milk against intestinal pathogens is shown in Figure 1. The four strains were able to inhibit the human intestinal pathogens *S. enterica* ATCC 13076 and *E. coli* ATCC 25922. The four strains were equally effective in the inhibition of *S. enterica* ATCC 13076; however, the TUCO-L5 strain showed the highest inhibition of *E. coli* ATCC 25922. Additionally, the four TUCO strains were able to inhibit the growth of the porcine pathogens enterotoxigenic (ETEC) *E. coli* TUCO-I5, enterohemorragic (EHEC) *E. coli* TUCO-I6, and *Salmonella* Typhimurium TUCO-I7 (Figure 1). The four strains were equally effective inhibitors of EHEC. The TUCO-L1 and TUCO-L5 strains had lower ability to inhibit ETEC and *Salmonella* Typhimurium, respectively, compared to that of the other strains isolated from the llama milk (Figure 1). Neutralization of acid did not reduce inhibition of the pathogen growth by llama milk strains indicating that other factors rather than lactic acid are responsible for the beneficial effect (data not shown).

**Figure 2:**
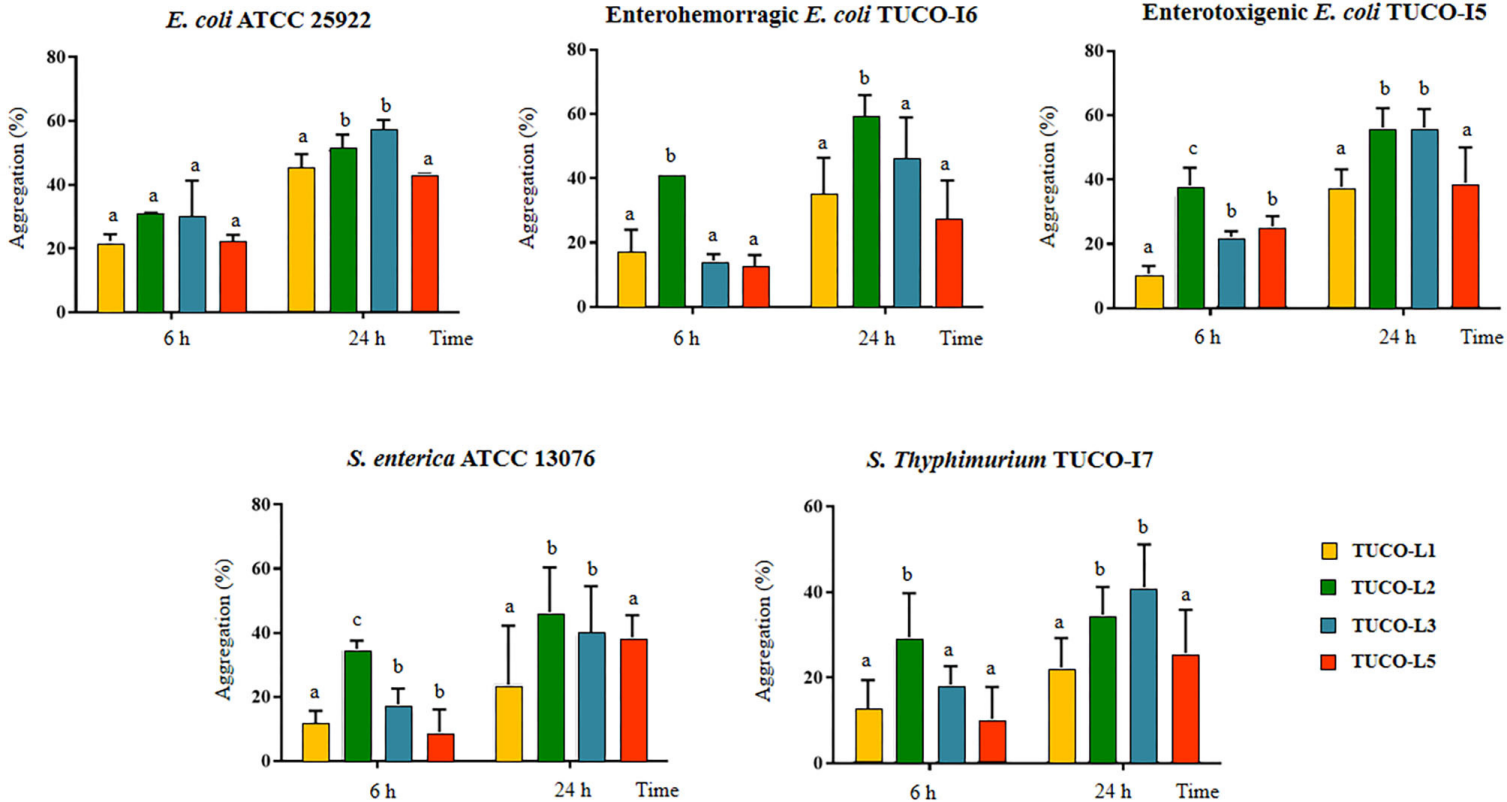
Coaggregation of Gram-negative bacterial pathogens with lactic acid bacteria (LAB) isolated from llama (*Lama glama*) milk. Coaggregate of llama milk strains of lactobacilli (TUCO-L2 and TUCO-L5) and enterococci (TUCO-L1 and TUCO-L3) with the pathogens was evaluated using the human intestinal pathogens *Salmonella enterica* ATCC 13076 and *Escherichia coli* ATCC 25922 and the porcine pathogens enterotoxigenic *E. coli* TUCO-I5, enterohemorragic *E. coli* TUCO-I6, and *Salmonella* Typhimurium TUCO-I7 at 6 and 24 h. The results represent the data of three independent experiments. Letters indicate significant differences (*P* < 0.05), a < b < c.

**Figure 3:**
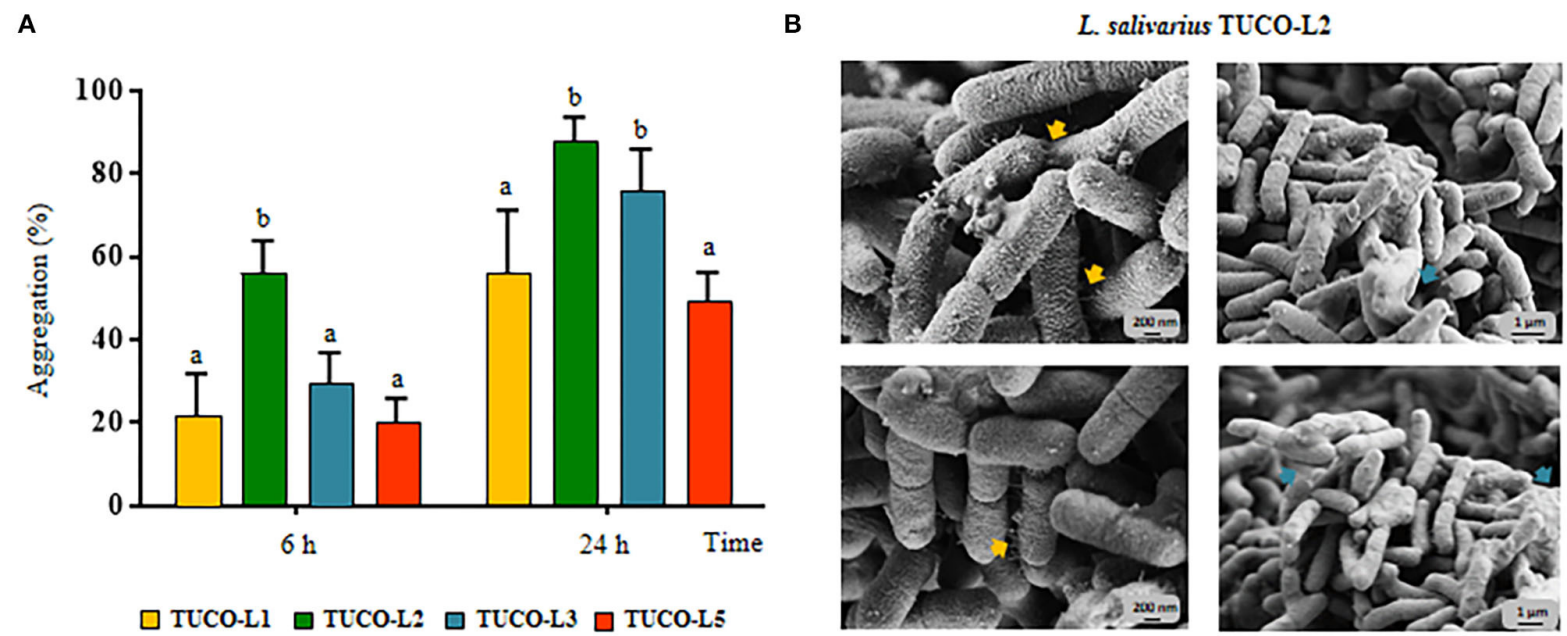
Autoaggregation of lactic acid bacteria (LAB) isolated from llama (*Lama glama*) milk. **(A)** Autoaggregation at 6 and 24 h of llama milk lactobacilli (TUCO-L2 and TUCO-L5) and enterococci (TUCO-L1 and TUCO-L3). The results represent the data of three independent experiments. Letters indicate significant differences (*P* < 0.05), a < b < c. **(B)** Scanning electron microscopy (SEM) images of aggregates of *Ligilactobacillus salivarius* TUCO-L2 showing putative exopolysaccharide (blue arrows) and fibril-like structures (yellow arrows).

The ability of the TUCO strains to coaggregate with intestinal pathogens was investigated. The TUCO-L2 and TUCO-L3 strains had the highest coaggregation with the tested intestinal pathogens (Figure 2). The ability of the TUCO-L2 strain to coaggregate with intestinal pathogens was substantially higher than that of other strains from llama milk at 6 h incubation. Similarly, autoaggregation of the TUCO-L2 and TUCO-L3 strains was higher than that of other strains (Figure 3A). The TUCO-L2 strain had the highest autoaggregation than that of other LAB strains at 6 h. Then, the TUCO-12 strain was used for SEM analysis. The microscopic observations demonstrated that the bacterial cells are bound by two clearly visible structures: short fibril-like structures that connect the cells to each other and the extracellular matrix that forms a cement surrounding the cells (Figure 3B). These structures may account for high aggregation of TUCO-L2 cells in the broth media and formation of rigid colonies in the agar media (Supplementary Figure 1).

Thus, the TUCO-L2 strain was selected for subsequent experiments. The complete genome of the TUCO-L2 strain was sequenced (accession number SOPE01000000), and the bacterium was identified as *Ligilactobacillus salivarius* (basonym: *Lactobacillus salivarius*) (Zheng et al., 2020). Therefore, the strain was designated as *L. salivarius* TUCO-L2 (Albarracin et al., 2020b).

### *In vitro* Adhesive and Immunomodulatory Properties of *L. salivarius* TUCO-L2

**Figure 4:**
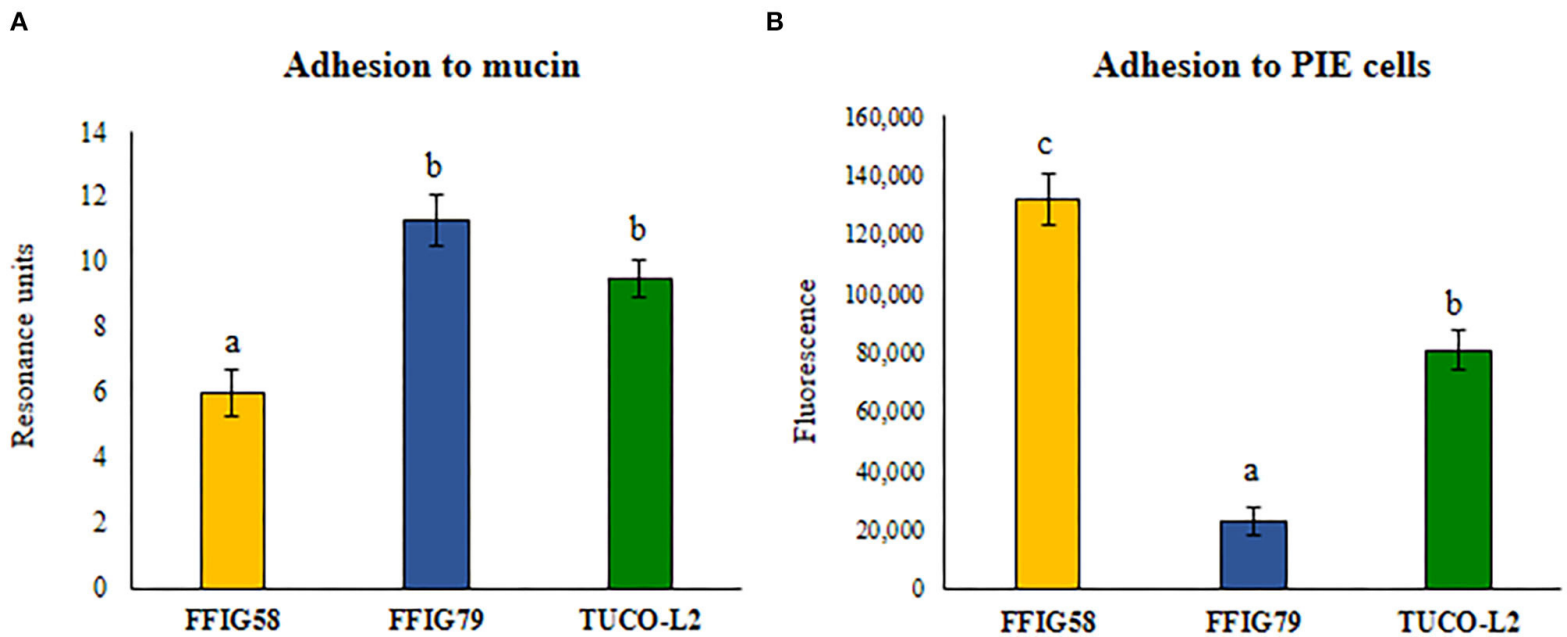
Adhesion of *Ligilactobacillus salivarius* TUCO-L2 isolated from llama (*Lama glama*) milk to porcine mucin and epithelial cells. The adhesion of the TUCO-L2 strain to porcine mucins and porcine intestinal epithelial (PIE) cells was compared with the porcine *L. salivarius* strains FFIG58 and FFIG79. Letters indicate significant differences (*P* < 0.05), a < b < c.

Then, the adhesion of *L. salivarius* TUCO-L2 to porcine mucins was evaluated. Porcine mucin from the small intestine was purified as described in Materials and Methods and used to evaluate the adhesion of the TUCO-L2 strain (Figure 4A). *L. salivarius* FFIG58 and FFIG79 isolated from the intestinal tract of wakame-fed pigs were used for comparison; these strains have been demonstrated to have low and high binding to porcine mucins, respectively (Zhou et al., 2020). *L. salivarius* TUCO-L2 demonstrated strong binding to porcine mucins that was not different from the binding of the FFIG79 strain (Figure 4A). Additionally, the ability of *L. salivarius* TUCO-L2 to adhere to porcine intestinal epithelial (PIE) cells was assessed. Comparison of the TUCO-L2 strain with the FFIG58 and FFIG79 strains, which have been demonstrated to have high and low adhesion to PIE cells, respectively (Zhou et al., 2020) indicated that *L. salivarius* TUCO-L2 adheres to PIE cells, and the adhesion was significantly different from that of the two control strains (Figure 4B).

**Figure 5:**
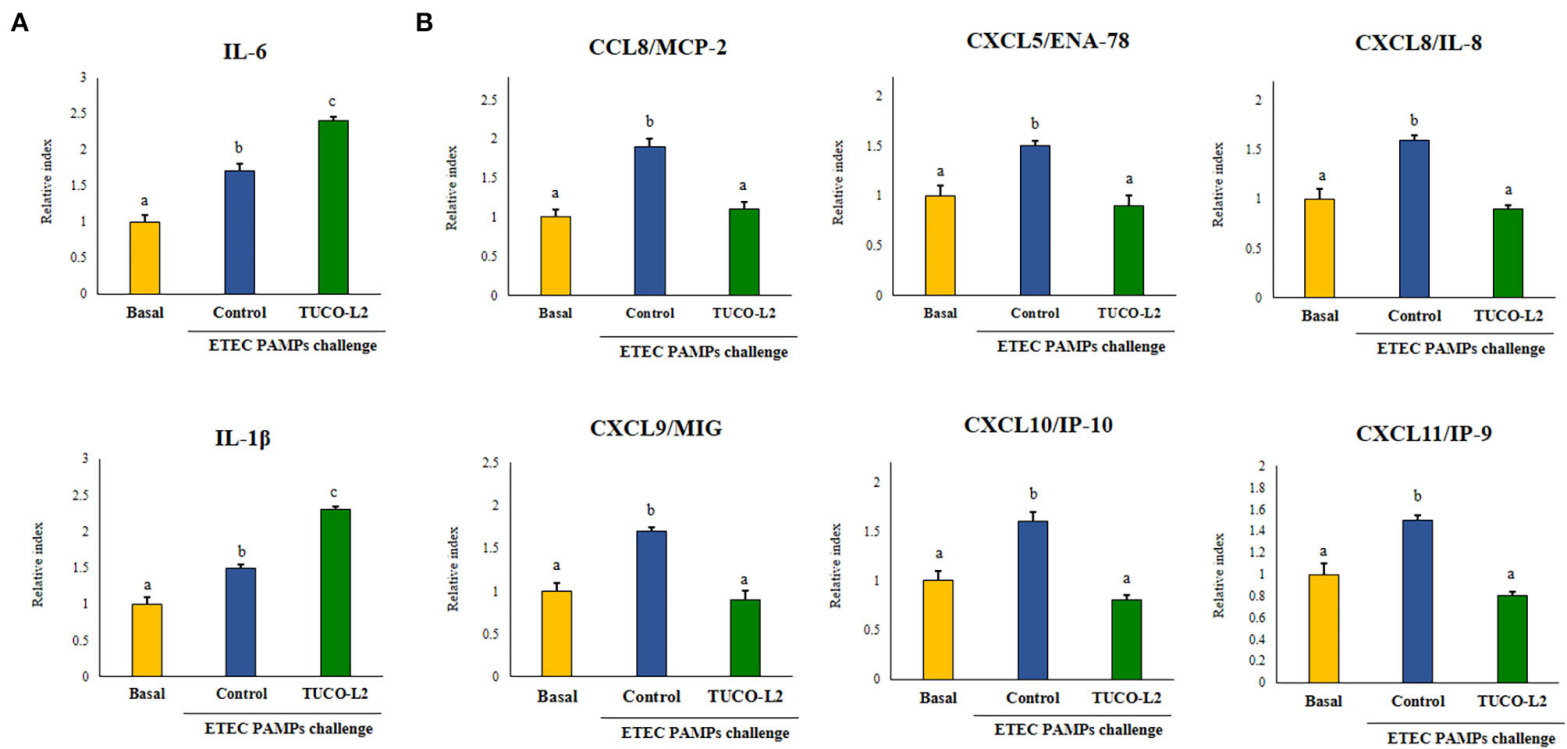
Immunomodulatory activity of *Ligilactobacillus salivarius* TUCO-L2 isolated from llama (*Lama glama*) milk in porcine intestinal epithelial (PIE) cells. PIE cells were stimulated with the TUCO-L2 strain and then challenged with pathogen-associated molecular patterns (PAMPs) from enterotoxigenic *Escherichia coli* to induce the activation of Toll-like receptor (TLR)-4. The expression levels of the cytokines **(A)** and chemokines **(B)** were assayed by RT-PCR 12 h after the ETEC PAMPs challenge. Letters indicate significant differences (*P* < 0.05), a < b < c.

The ability of *L. salivarius* TUCO-L2 to differentially modulate the innate immune response in PIE cells was evaluated by triggering the activation of TLR4 in lactobacilli-treated epithelial cells (Figure 5). PIE cells challenged with ETEC PAMPs in the absence of lactobacilli were used as a control. As reported previously, TLR4 activation by ETEC PAMPs resulted in an increase in the expression of proinflammatory cytokines *IL-1*β and *IL-6* in PIE cells compared to cells that were not stimulated with ETEC PAMPs (basal group) (Shimazu et al., 2012; Garcia-Castillo et al., 2019). *L. salivarius* TUCO-L2 significantly increased the levels of both inflammatory cytokines compared to those in the control (Figure 5A). The expression levels of proinflammatory chemokines *CCL8, CXCL5, CXCL8, CXCL9, CXCL10*, and *CXCL11* were increased in ETEC PAMPs-treated PIE cells after TLR4 activation. *L. salivarius* TUCO-L2 significantly reduced the expression of these chemokines compared to those in the ETEC PAMPs-challenged control PIE cells (Figure 5B).

**Figure 6:**
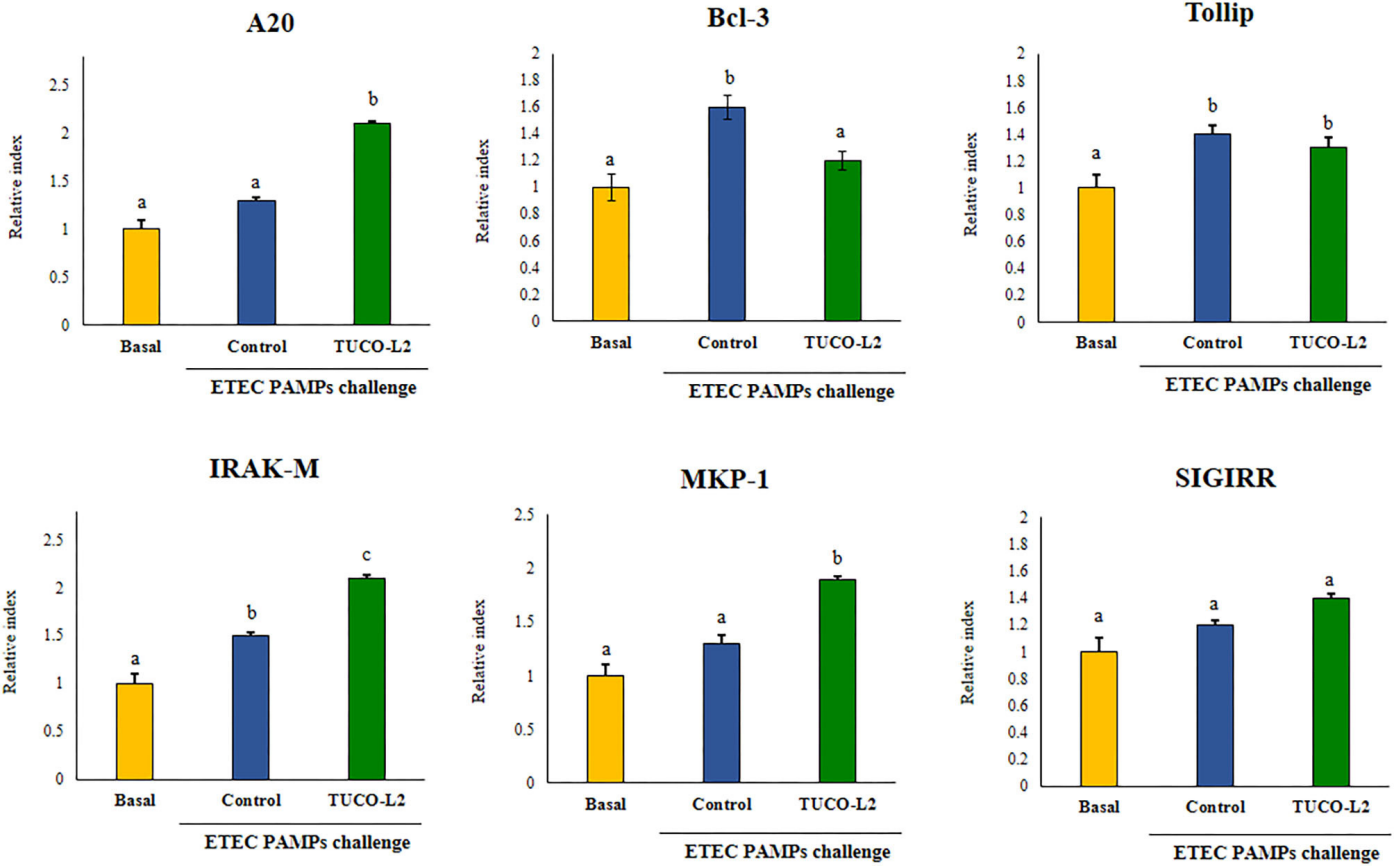
Immunomodulatory activity of *Ligilactobacillus salivarius* TUCO-L2 isolated from llama (*Lama glama*) milk in porcine intestinal epithelial (PIE) cells. PIE cells were stimulated with the TUCO-L2 strain and then challenged with pathogen-associated molecular patterns (PAMPs) from enterotoxigenic *Escherichia coli* to induce the activation of Toll-like receptor (TLR)-4. The expression levels of the negative regulators of the TLR signaling pathway were evaluated by RT-PCR 12 h after the ETEC PAMPs challenge. Letters indicate significant differences (*P* < 0.05), a < b < c.

Differential modulation of the expression of the negative regulators of the TLR4 signaling pathway by *L. salivarius* TUCO-L2 was assessed in PIE cells (Figure 6). The treatment of PIE cells with ETEC PAMPs significantly augmented the expression of *A20, Bcl-3, Tollip*, and *IRAK-M*, while the expression levels of *MKP-1* and *SIGIRR* were not modified compared to those in the unchallenged control cells (basal group). The treatment of PIE cells with *L. salivarius* TUCO-L2 significantly increased the expression of *A20, IRAK-M*, and *MKP-1* compared to those in the ETEC PAMPs-challenged control PIE cells (Figure 6). The expression level of *Bcl-3* in TUCO-L2-treated PIE cells was significantly lower than that in the ETEC PAMPs-challenged control PIE cells.

### General Genomic Features of *L. salivarius* TUCO-L2

The complete genome of *L. salivarius* TUCO-L2 was sequenced by Illumina HiSeq, annotated, and recently published (Albarracin et al., 2020b) to provide detailed characterization of the probiotic/immunobiotic properties. The genomic analysis of the TUCO-L2 strain was performed by comparison with publicly available genomes of *L. salivarius* strains isolated from human milk (LPM01 and CECT5713), human intestinal tract (UCC118 and REN), porcine intestine (JCM1046 and ZSL006), and chicken intestine (DJ-sa-01 and CICC23174). The general genomic features of *L. salivarius* strains used in the present study are summarized in the Supplementary Table 2. The TUCO-L2 draft genome sequence has an average GC content of 33%, a total estimated size of 1,600,747 bp, and 1,691 protein-coding genes. These general genomic features were similar to those of the *L. salivarius* strains used for comparison. However, the TUCO-L2 strain had a smaller genome size compared with that of other *L. salivarius* strains. Their genome size varied from 1,746,897 bp in the chicken strain CICC 23174 to 2,177,581 bp in the porcine strain ZLS006 (Supplementary Table 2). The number of protein-coding genes in the TUCO-L2 strain was among the lowest of all strains, and only the CICC 23174 strain had lower number of protein-coding genes than that in *L. salivarius* TUCO-L2.

**Figure 7:**
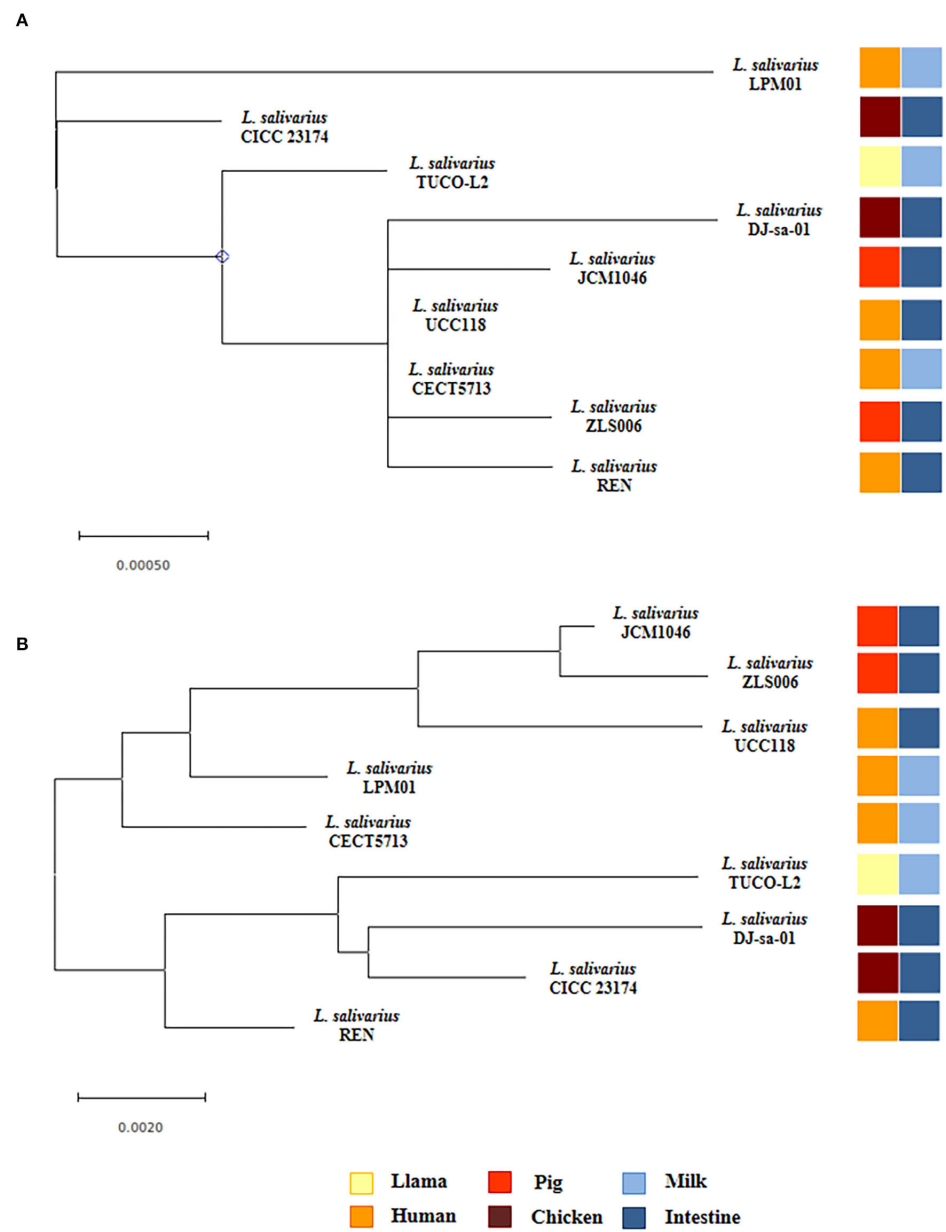
Hierarchical clustering of *Ligilactobacillus salivarius* TUCO-L2 isolated from llama (*Lama glama*) milk compared with the strains of the same species isolated from different origins. **(A)** Phylogenetic tree constructed using 16s RNA extracted from the genome of TUCO-L2 strain and from the publicly available complete genomes of the *L. salivarius* strains isolated from human milk (LPM01 and CECT5713), human intestinal tract (UCC118 and REN), porcine intestine (JCM1046 and ZSL006), and chicken intestine (DJ-sa-01 and CICC23174). **(B)** Molecular phylogenetic analysis by maximum likelihood method of MLST of *L. salivarius* strains. The phylogenetic trees were constructed based on the MLST analysis by using the *parB, rpsB, pheS, nrdB, groEL*, and *ftsQ* genes present the genomes of the TUCO-L2 strain and publicly available genomes of the *L. salivarius* strains.

Phylogenetic trees were constructed based on the sequences of the 16s rRNA genes (Figure 7A) and the maximum likelihood analysis of MLST of the sequences of the *parB, rpsB, pheS, nrdB, groEL*, and *ftsQ* genes (Figure 7B) (Harris et al., 2017; Lee et al., 2017). Both methods of analysis indicated that *L. salivarius* TUCO-L2 clustered separately from other evaluated strains. Notably, the MLST analysis indicated that the TUCO-L2 strain is clustered close to the two strains originally isolated from the chicken intestine (DJ-sa-01 and CICC23174).

**Figure 8:**
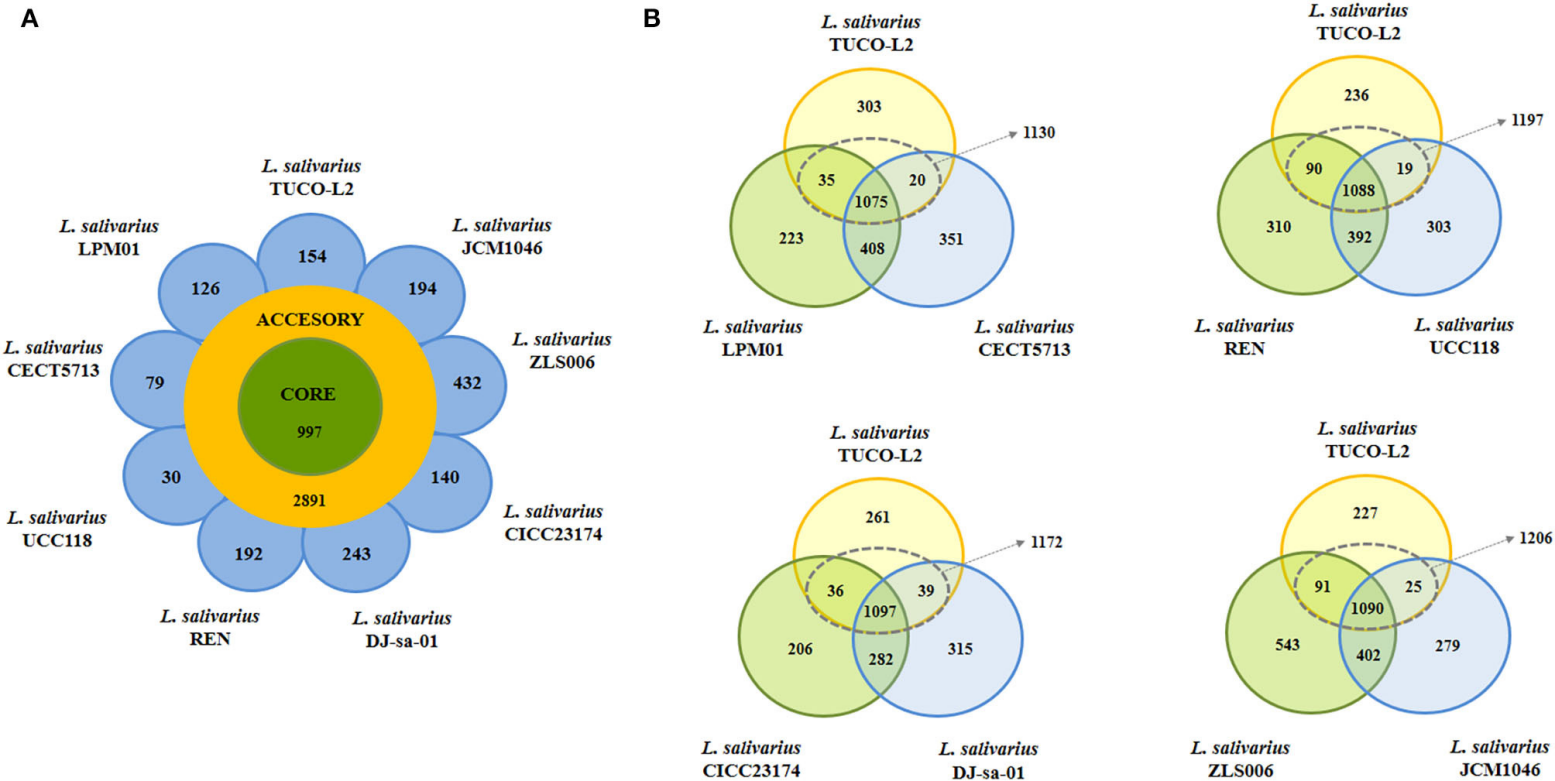
Genomic comparison of *Ligilactobacillus salivarius* TUCO-L2 isolated from llama (*Lama glama*) milk with the strains of the same species isolated from various sources. **(A)** Venn diagram of the number of genes in the core genome and accessory genome and unique genes in the genome of TUCO-L2 strain and in the genomes of *L. salivarius* strains isolated from human milk (LPM01 and CECT5713), human intestinal tract (UCC118 and REN), porcine intestine (JCM1046 and ZSL006), and chicken intestine (DJ-sa-01 and CICC23174). **(B)** Venn diagrams comparing *L. salivarius* TUCO-L2 with various strains based on the origin of the strains.

Comparison of the complete genomes of *L. salivarius* TUCO-L2 and the control strains revealed a core genome of 997 genes and an accessory genome of 2,891 genes (Figure 8A). The TUCO-L2 strain has 154 unique genes that are not shared with other strains. However, this number was not the highest number of unique genes since the ZLS006, DJ-sa-01, JCM1046, and REN strains had 432, 243, 194, and 192 unique genes, respectively. Unique genes of the TUCO-L2 strain included genes of the inositol catabolism pathway [methylmalonate semialdehyde dehydrogenase (*iolA*), 5-deoxy-glucuronate isomerase (*iolB*), 3D-(3,5/4)-trihydroxycyclohexane-1,2-dione hydrolase (*iolD*), inosose dehydratase (*iolE*), inositol 2-dehydrogenase/D-chiro-inositol 3-dehydrogenase (*iolG*), and inosose isomerase (*iolI*)]. The genes encoding for the iron-sulfur cluster assembly were also detected, including PaaD-like protein, cysteine desulfurase, iron-sulfur cluster assembly proteins SufB, SufE2, and SufD, and iron-sulfur cluster assembly ATPase protein SufC. Unique genes of the TUCO-L2 strain encode for a high affinity phosphate transporter and control of PHO regulon, including a phosphate ABC transporter, periplasmic phosphate-binding protein (*PstS*), phosphate transport ATP-binding protein (*PstB*), and phosphate transport system permease proteins PstA (*PstA*) and PstC (*PstC*), and components of the common pathway for the synthesis of aromatic compounds (3-dehydroquinate dehydratase I and shikimate/quinate 5-dehydrogenase I beta) (Supplementary Tables 3–6).

Subsequent comparison accounted for different origins of the control strains (Figure 8B). Genomic comparison of *L. salivarius* TUCO-L2 with the human milk isolates revealed that the llama milk strain shares a total of 1,130 genes with the CECT 5713 and LPM01 strains and has 303 unique genes. Comparison with the CECT 5713 and LPM01 strains isolated from human milk indicated that unique genes of the *L. salivarius* TUCO-L2 genome (Supplementary Table 3) include genes of the accessory SecA2-SecY2 system (*secA2, secY2, asp1, asp2*, and *asp3*), exopolysaccharide (EPS) biosynthesis (*cap8A, epsE1, epsE2, epsJ1-J5*, and *epsL*), glycosyltransferases (*gtf1, gtf2, gtfC, wfgD, wbbI2*, and *wbbI3)*, glucosidases (*bglH1, bglH2*, and *bglK*), and choloylglycine hydrolase (*cbh1*). Similar results were obtained by comparison of the *L. salivarius* TUCO-L2 genome with the genomes of the UCC118 and REN strains with the exception of the *cbh1* gene that was also detected in the human intestinal strains (Supplementary Table 4).

Comparison with the JCM1046 and ZLS006 strains isolated from porcine intestine (Supplementary Table 5) and the DJ-sa-01 and CICC23174 strains isolated from chicken intestine (Supplementary Table 6) identified the following unique genes in the *L. salivarius* TUCO-L2 genome: EPS biosynthesis (*cap8A, epsE1, epsE2, epsJ1-J5*, and *epsL*), glycosyltransferases (*gtf1, gtf2, gtfC, wfgD, wbbI2*, and *wbbI3*), and glucosidases (*bglH1, bglH2*, and *bglK*). In contrast to human strains, the genes of the accessory SecA2-SecY2 system (*secA2, secY2, asp1, asp2*, and *asp3*) were detected in all *L. salivarius* strains of animal origin. Notably, the *cbh1* gene of the TUCO-L2 strain was not detected in the genomes of the strains isolated from chicken intestine.

### Genomic Analysis of Potential Probiotic Molecules in *L. salivarius* TUCO-L2

**Figure 9:**
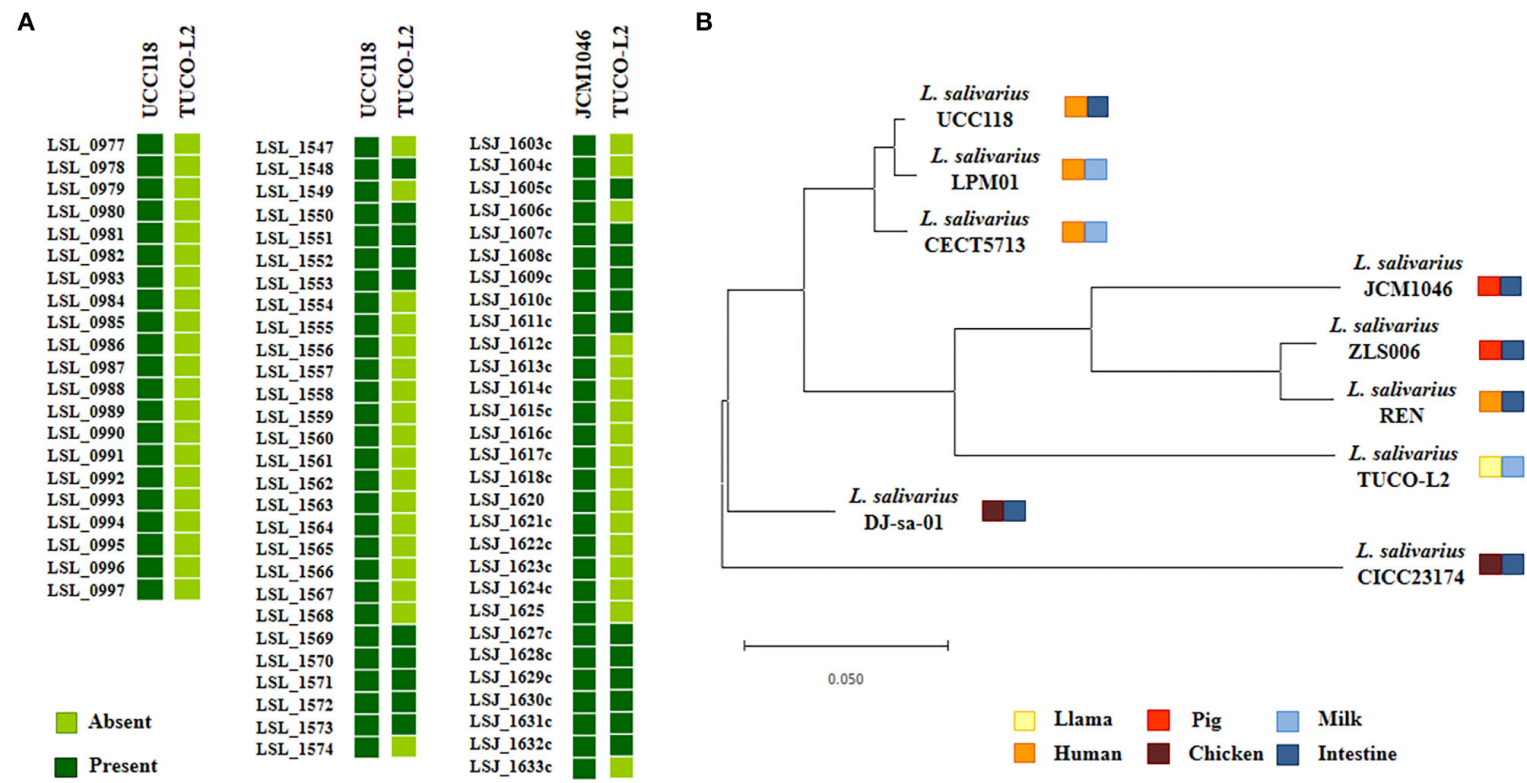
Genomic comparison of the exopolysaccharides (EPS) cluster of *Ligilactobacillus salivarius* TUCO-L2 isolated from llama (*Lama glama*) milk with that of the strains of the same species. **(A)** The EPS clusters from *L. salivarius* UCC118 (EPS1: from LSL_077 to LSL_097; EPS2: from LSL_1547 to LSL_1574) and JCM1046 (EPS3: from LSJ_1603c to LSJ_1633c) were used for comparison. **(B)** Phylogenetic tree was constructed by using the sequences of twelve conserved EPS genes shared by the strains isolated from human milk (LPM01 and CECT5713), human intestinal tract (UCC118 and REN), porcine intestine (JCM1046 and ZSL006), chicken intestine (DJ-sa-01 and CICC23174), and llama milk (TUCO-L2).

Considering that some of the genes specific for *L. salivarius* TUCO-L2 are involved in EPS biosynthesis, the EPS-related genes in the genomes of the TUCO-L2 and *L. salivarius* strains were compared. The EPS clusters described in the *L. salivarius* strains UCC118 (Harris et al., 2017) and JCM1046 (Raftis et al., 2011) were used as a reference. Two EPS gene clusters were detected in *L. salivarius* UCC118, including EPS cluster 1 containing 20 genes and EPS cluster 2 containing 27 genes, and a single EPS gene cluster containing 28 genes was detected in *L. salivarius* JCM1046 (Figure 9A). The EPS cluster of the JCM1046 strain was designated as EPS cluster 3 in this study. None of the genes of EPS cluster 1 were detected in the genome of *L. salivarius* TUCO-L2. On the other hand, the conserved genes of EPS clusters 2 and 3 (Raftis et al., 2011; Harris et al., 2017) were detected in the TUCO-L2 strain, including undecaprenyl-phosphate β-glucose phosphotransferase, transcriptional regulator LytR, a phosphotyrosine protein phosphatase, a tyrosine protein kinase, a chain length regulator, β-N-acetylhexosaminidase, dTDP-4-dehydrorhamnose reductase, dTDP-glucose 4,6-dehydratase, dTDP-4-dehydrorhamnose 3,5-epimerase, and glucose-1-phosphate thymidylyltransferase (Figure 9A). The gene LSL_1549 (glycosyltransferase) and the genes LSL_1555 to LSL_1565 mainly corresponding to glycosyltransferases and transposases were not detected in the genome of the TUCO-L2 strain. Similar results were obtained when EPS cluster 3 was used to analyze the TUCO-L2 genome. These results were anticipated since EPS clusters 2 and 3 have substantial similarities (Raftis et al., 2011; Harris et al., 2017). Interestingly, the genes LSJ_1604c, LSJ_1606c, and LSJ_1633c that encode for glycosyltransferases in EPS cluster 3 were not detected in the TUCO-L2 genome. Analysis of the conserved genes in EPS clusters 2 and 3 identified 12 EPS core genes that were used for subsequent analysis of EPS in all genomes of the *L. salivarius* strains. The phylogenetic tree constructed based on the sequences of 12 EPS core genes demonstrated that the TUCO-L2 strain is clustered separately from other *L. salivarius* strains (Figure 9B).

**Figure 10:**
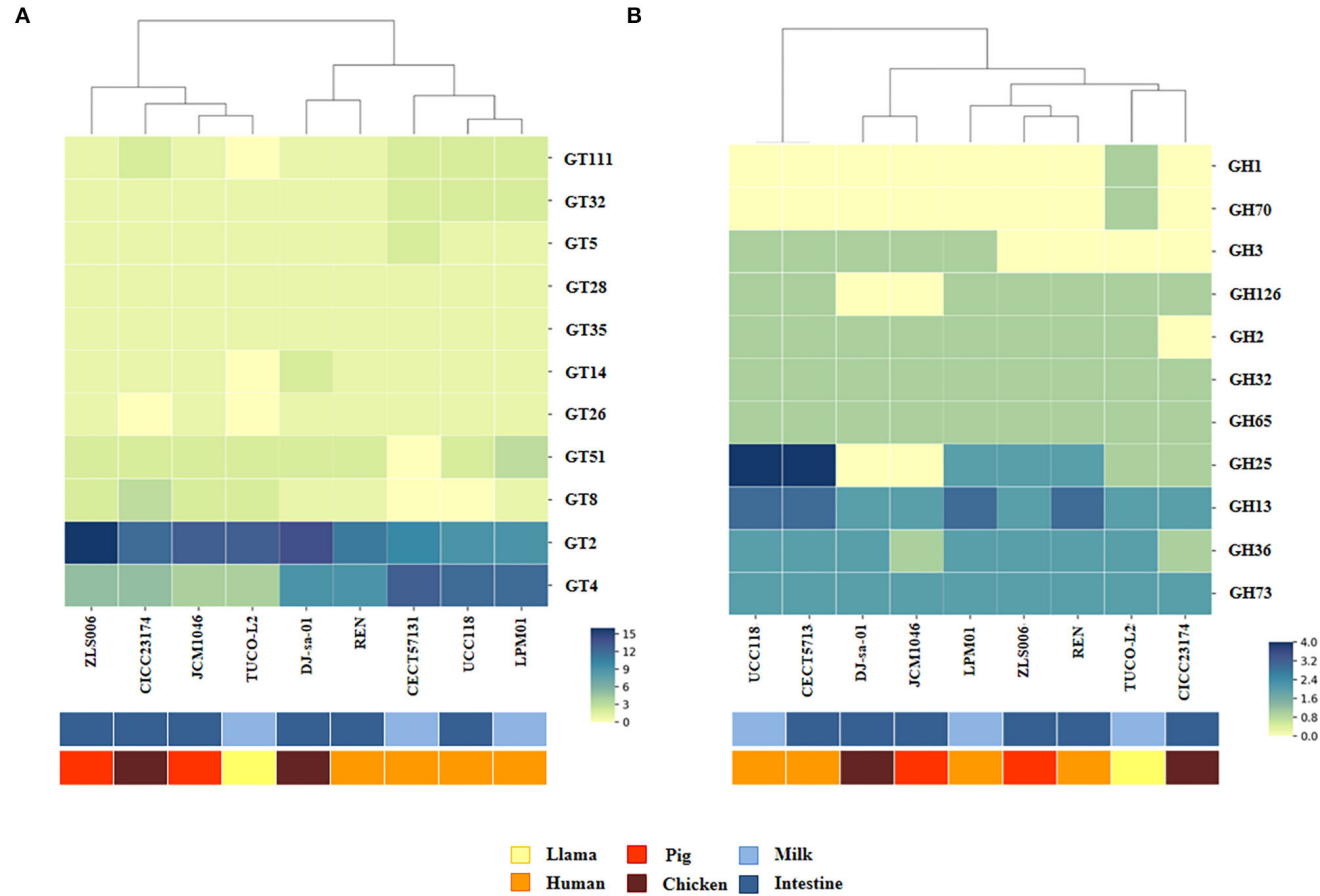
Genomic comparison of glycosyltransferases and glycosylhydrolases of *Ligilactobacillus salivarius* TUCO-L2 isolated from llama (*Lama glama*) milk with those of the strains of the same species isolated from different origins. Numbers of glycosyltransferases **(A)** and glycosylhydrolases **(B)** identified in the genomes of TUCO-L2 strain and in the genomes of *L. salivarius* strains isolated from human milk (LPM01 and CECT5713), human intestinal tract (UCC118 and REN), porcine intestine (JCM1046 and ZSL006), and chicken intestine (DJ-sa-01 and CICC23174). Heatmaps were constructed based on the numbers of glycosyltransferases or glycosylhydrolases in each family.

Various types of glycosyltransferases encoded by the genomes of EPS-producing bacteria can modify the structure of the EPS molecule and consequently change its functionality. Then, the abundance of the genes encoding for glycosyltransferase families in the TUCO-L2 strain was evaluated and compared with that in the other eight *L. salivarius* strains (Figure 10A). Interestingly, the results of the clustering analysis accounting for the number and type of glycosyltransferases showed that the TUCO-L2 strain is clustered separately from most of the other strains and is close only to the porcine intestinal JCM1046 strain. Comparison of glycosylhydrolases in the genomes of the *L. salivarius* strains (Figure 10B) indicated that the TUCO-L2 strain is clustered separately from most of the other strains and is close only to the chicken intestine CICC23174 strain. Notably, *L. salivarius* TUCO-L2 had significantly higher number of glycosylhydrolases of the GH1 and GH70 families.

The whole genome sequence of *L. salivarius* TUCO-L2 was analyzed to identify antimicrobial substance(s) responsible for the antipathogenic effect demonstrated in the present study. Therefore, the BAGEL 4 platform and blastp were used to search for bacteriocins in the TUCO-L2 genome. The analysis indicated the absence of any similarity to known bacteriocin genes. Thus, the whole genome sequence of TUCO-L2 was annotated to identify two genes encoding for a bacteriocin-prepeptide or an inducing factor for bacteriocin synthesis and bacteriocin immunity indicating the presence of a novel bacteriocin in this strain from llama milk (Supplementary Table 7).

**Figure 11:**
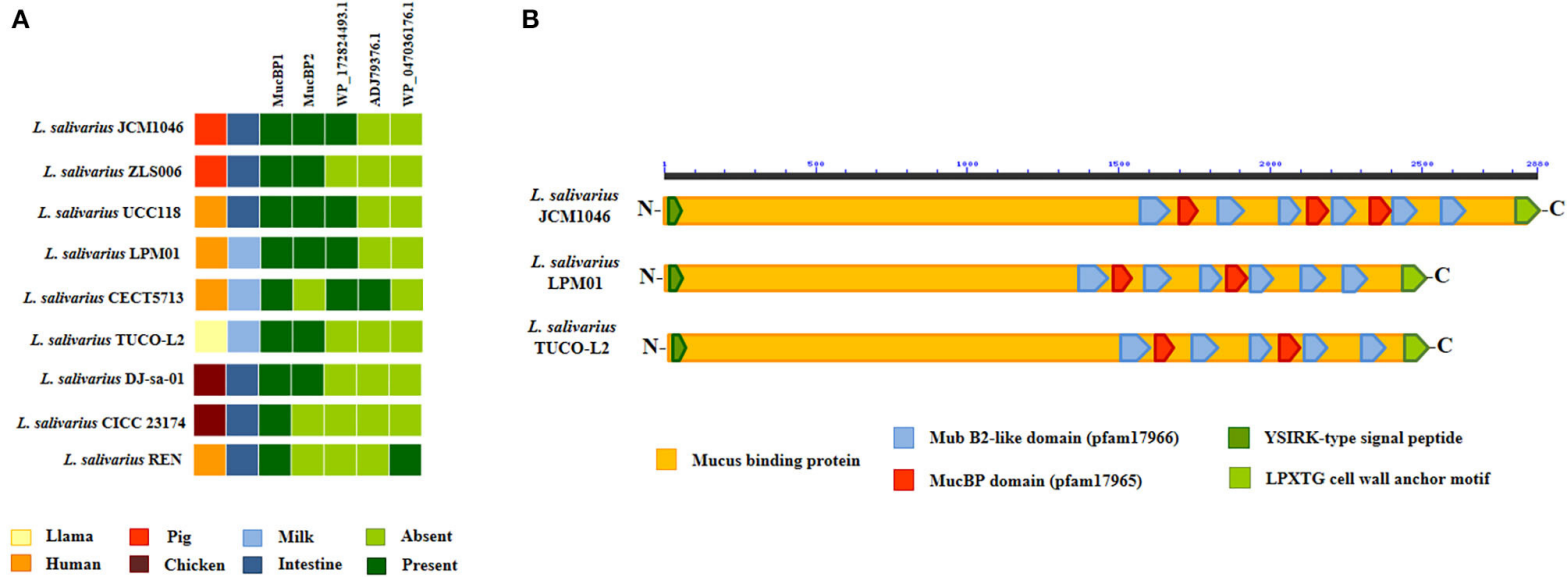
Genomic comparison of the mucus-binding proteins (MucBPs) of *Ligilactobacillus salivarius* TUCO-L2 isolated from llama (*Lama glama*) milk with the strains of the same species. **(A)** MucPBs genes of the TUCO-L2 strain were compared with those of the *L. salivarius* strains isolated from human milk (LPM01 and CECT5713), human intestinal tract (UCC118 and REN), porcine intestine (JCM1046 and ZSL006), and chicken intestine (DJ-sa-01 and CICC23174). **(B)** Functional domains present in MucBP2 of *L. salivarius* TUCO-L2 and compared with those of the proteins encoded by the genomes of the JCM1046 and LPM01 strains.

Comparative genomic analysis was used to characterize the adhesion factors of *L. salivarius* TUCO-L2 by investigating several proteins and systems that were described to be involved in the adhesion of lactobacilli to the intestinal mucosa. Several mucus-binding proteins (MucBP) have been identified in lactic acid bacteria and are associated with their ability to colonize the gastrointestinal tract (Latousakis and Juge, 2018). Then, we searched for MucBP genes in the genome of *L. salivarius* TUCO-L2 (Figure 11). As described previously (Lee et al., 2017), a common mucus-binding protein (designated here as MucBP1) was detected in all *L. salivarius* strains, including the TUCO-L2 strain. Four MucBP were detected in the genomes of the tested *L. salivarius* strains, and only a single MucBP gene WP_087118522.1 (designated here as MucBP2) was detected in the genome of the TUCO-L-2 strain (Figure 11A). This protein is similar but not identical to MucBP of the LPM01, UCC118, DJ-sa-01, JCM1046, and ZLS006 strains (Figure 11B).

**Figure 12:**
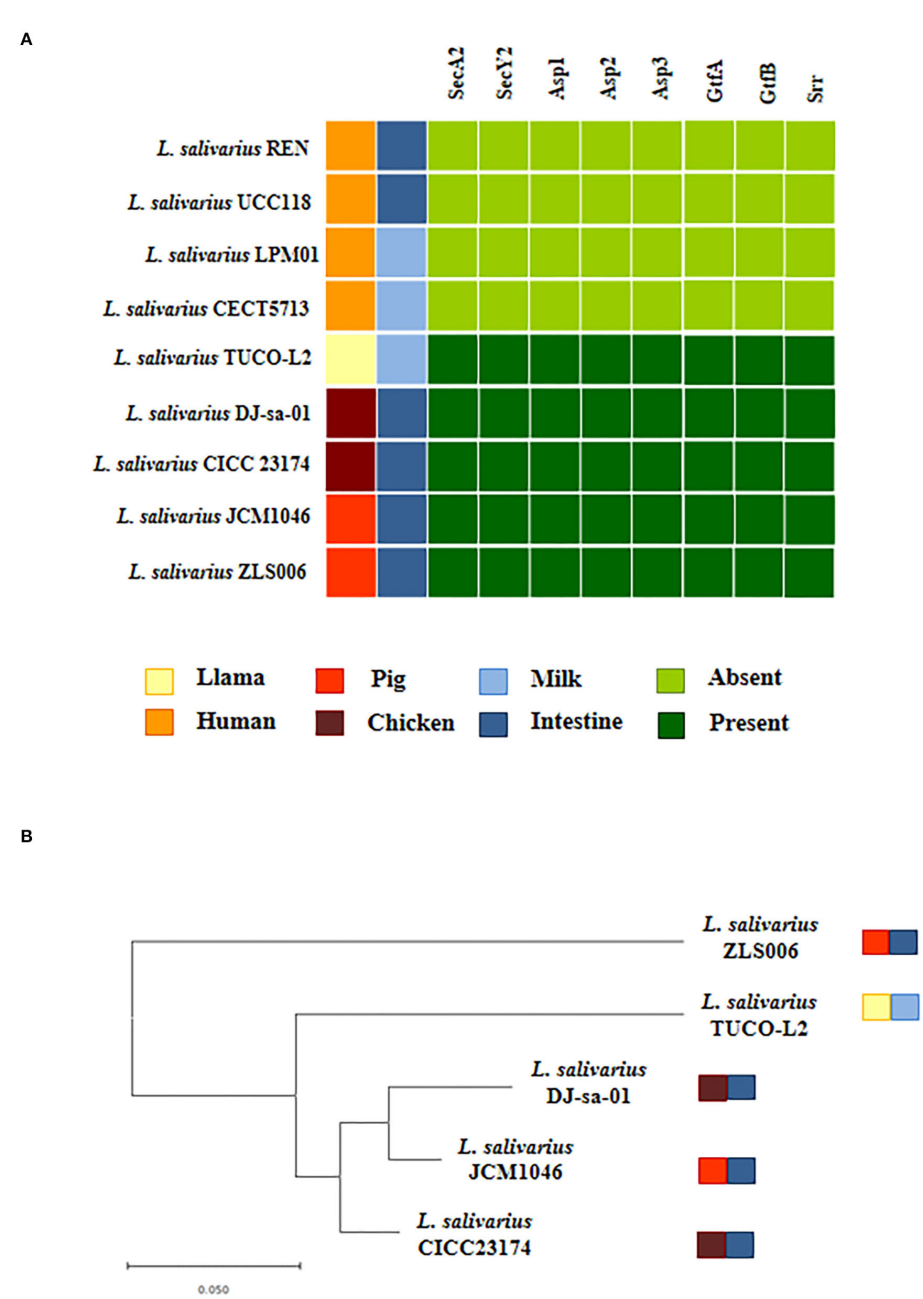
Genomic comparison of the SeA2-SecY2 accessory secretion system of *Ligilactobacillus salivarius* TUCO-L2 isolated from llama (*Lama glama*) milk with the strains of the same species. **(A)** The SecA2-SecY2 cluster of the TUCO-L2 strain compared with that of the *L. salivarius* strains isolated from human milk (LPM01 and CECT5713), human intestinal tract (UCC118 and REN), porcine intestine (JCM1046 and ZSL006), and chicken intestine (DJ-sa-01 and CICC23174). **(B)** Phylogenetic tree was constructed by using the sequences of the s*ecA2, secY2, asp1, asp2, asp3, gtfA*, and *gtfB* genes shared by the strains of animal origin.

Our data indicate that similar to other *L. salivarius* strains from animals, the TUCO-L2 strain possesses the genes for the accessory Sec system. The SecA2-SecY2 secretion system is associated with adhesion of certain lactobacilli strains to mucosal tissues (Frese et al., 2013; De Boeck et al., 2020). Then, the presence of this cluster was investigated by manual search for the genes encoding for motor protein SecA2, membrane translocation complex SecY2, chaperones Asp1-3, and glycosyltransferases GtfA and GtfB. Complete cluster was detected in the genome of *L. salivarius* TUCO-L2 and in the genomes of the strains isolated from animal intestine (Figure 12A). Moreover, in agreement with our previous results (Supplementary Tables 3, 4) and the data of other authors (Lee et al., 2017), the SecA2-SecY2 cluster was not detected in the human-related *L. salivarius* strains LPM01, CECT5713, UCC118, and REN that were used as a reference in the present study (Figure 12A). Notably, the *secA2, secY2*, and *gtfB* genes of the TUCO-L2 strain showed 99.8, 100, and 97.5% homology, respectively, with the similar genes of the porcine JCM1046 strain (Supplementary Figure 3). Additionally, the *asp1* and *asp3* genes showed 98.7% homology with the similar genes in the chicken DJ-sa-01 strain. Although all genomes of the *L. salivarius* strains of animal origin contained the genes of the SecA2-SecY2 system, our results of evaluation of the phylogenetic clustering based on the nucleotide sequences of the conserved *secA2, secY2, asp1, asp2, asp3, gtfA*, and *gtfB* genes indicate some degree of divergence (Figure 12B).

Then, the presence of accessory Sec-dependent serine-rich glycoprotein adhesion proteins or srr adhesins was assessed within the SecA2-SecY2 system cluster of the TUCO-L2 strain. We detected the presence of two partial sequences of srr proteins: WP_134354846.1 and WP_134354365.1. Both sequences contained the KxYKxGKxW signal peptide, N-terminal serine-rich repeat adhesion glycoprotein AST domain, and partial regions of serine-rich repeats (data not shown). The blast analysis of these two sequences revealed a 45.6% identity with a 59% query cover suggesting that the sequences represent two different srr adhesins. These putative adhesins WP_134354846.1 and WP_134354365.1 were designated srr1 and srr2, respectively. Unfortunately, due to the fragmentation of the contigs, we are unable to identify the C terminal regions of these two putative adhesins.

Pili are involved in the intestinal colonization of lactobacilli associated with probiotic effects (Kankainen et al., 2009). Previous genomic studies by Harris et al. (2017) showed that only 5 of 43 *L. salivarius* genomes contained the genes encoding for extra sortase A, sortase C, and putative pilin subunits. These strains include *L. salivarius* JCM1047, DSM20555, ATCC11741, gul1, and gul2. Additional search of the NCBI genome bank for other *L. salivarius* strains with a pili operon detected this cluster only in the genome of *L. salivarius* A3iob originally isolated from the bee intestine (Audisio et al., 2018). Then, the pilus operon of these strains was compared with that of *L. salivarius* TUCO-L2 (Supplementary Figure 3). The genes encoding for extra sortase A, sortase C, or pilin subunits were not detected in the genome of the TUCO-L2 strain.

### *In vivo* Anti-salmonella Effect of *L. salivarius* TUCO-L2

**Figure 13:**
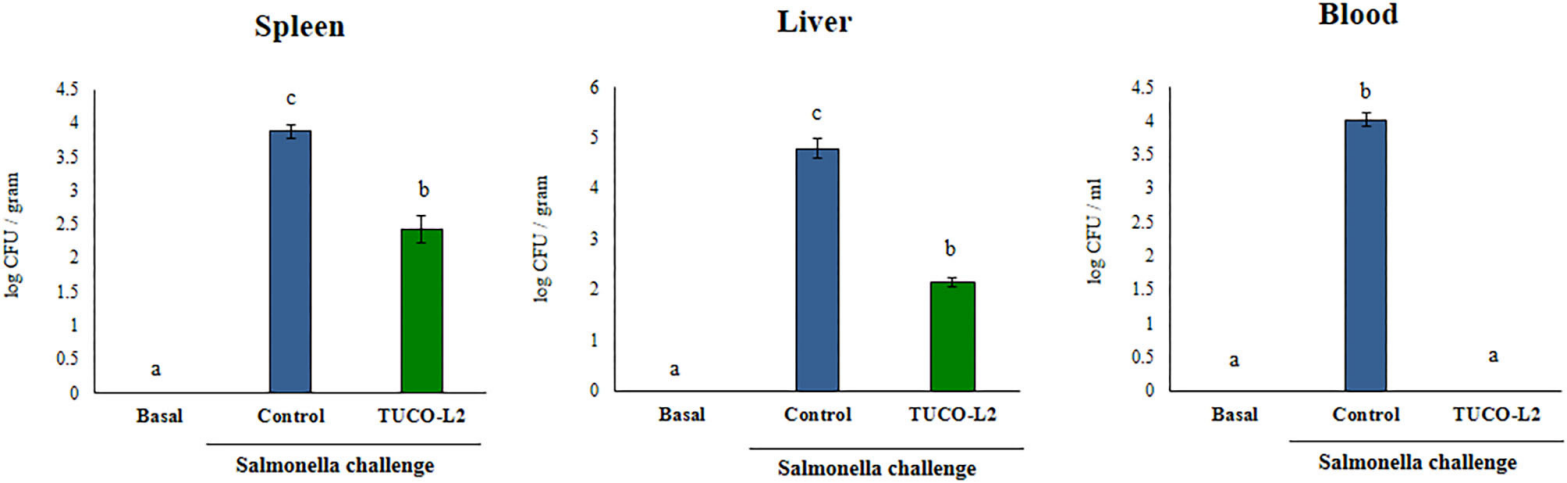
The effect of *Ligilactobacillus salivarius* TUCO-L2 isolated from llama (*Lama glama*) milk on the resistance of adult mice to *Salmonella* infection. *L. salivarius* TUCO-L2 was administered to male 6-week-old Balb/c mice for 5 consecutive days at a dose of 10^8^ cells/mouse/day in drinking water. Untreated mice were used as a control. TUCO-L2-treated and control mice were challenged by oral administration of 10^7^ cells/mouse of *S. typhimurium* (20LD50) on day 6. Mice were sacrificed on day 2 after the infection, and the bacterial pathogen counts were determined in the liver, spleen, and blood of mice. Untreated and uninfected mice were used for comparison (basal group). Each parameter was assayed in 5–6 mice per group. Letters indicate significant differences (*P* < 0.05), a < b < c.

The *in vitro* and *in silico* data indicated the beneficial effects of the TUCO-L2 strain in the protection against intestinal pathogens. Thus, we performed *in vivo* studies to demonstrate the probiotic potential of this strain. The ability of *L. salivarius* TUCO-L2 to reduce *Salmonella* infection was studied in a mouse model (Figure 13). The TUCO-L2 strain was administered to mice for 5 consecutive days at a dose of 10^8^ cells/mouse/day in drinking water, and the animals were orally challenged with pathogenic *Salmonella* Typhimurium as described previously (Quilodrán-Vega et al., 2016). The administration of *L. salivarius* TUCO-L2 induced a significant decrease in the number of *Salmonella* in the liver and spleen of treated mice compared to that in the control animals (Figure 13). Moreover, the pathogen was not detected in the blood of animals treated with the TUCO-L2 strain (Figure 13).

**Figure 14:**
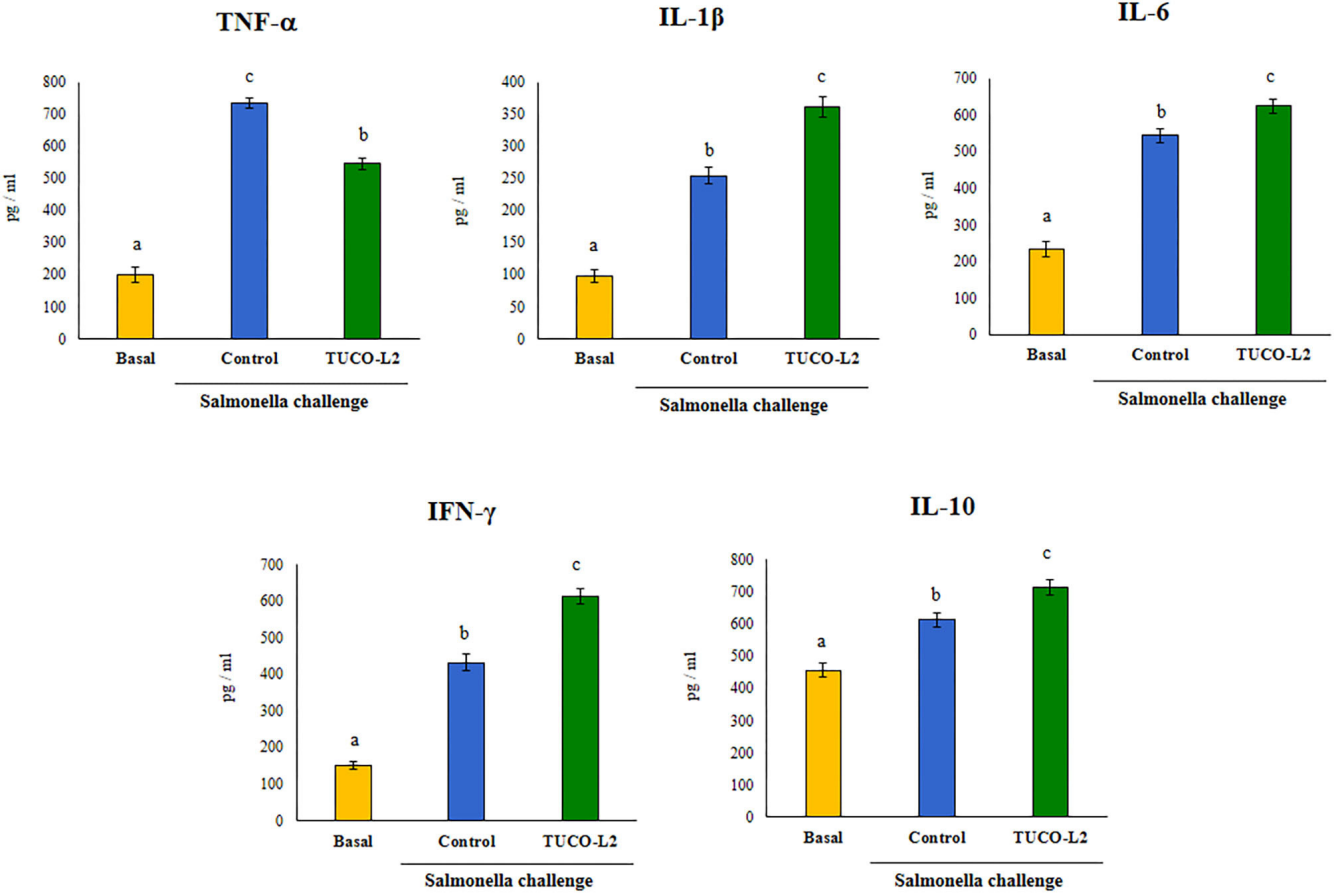
The effect of *Ligilactobacillus salivarius* TUCO-L2 isolated from llama (*Lama glama*) milk on the intestinal immune response of adult mice to *Salmonella* infection. *L. salivarius* TUCO-L2 was administered to male 6-week-old Balb/c mice for 5 consecutive days at a dose of 10^8^ cells/mouse/day in drinking water. Untreated mice were used as a control. TUCO-L2-treated and control mice were challenged by oral administration of 10^7^ cells/mouse of *S. typhimurium* (20LD50) on day 6. Mice were sacrificed on day 2 after the infection, and the levels of intestinal TNF-α, IFN-γ, IL-1β, IL-6, and IL-10 were measured by ELISA. Untreated and uninfected mice were used for comparison (basal group). Each parameter was assayed in 5–6 mice per group. Letters indicate significant differences (*P* < 0.05), a < b < c.

**Figure 15:**
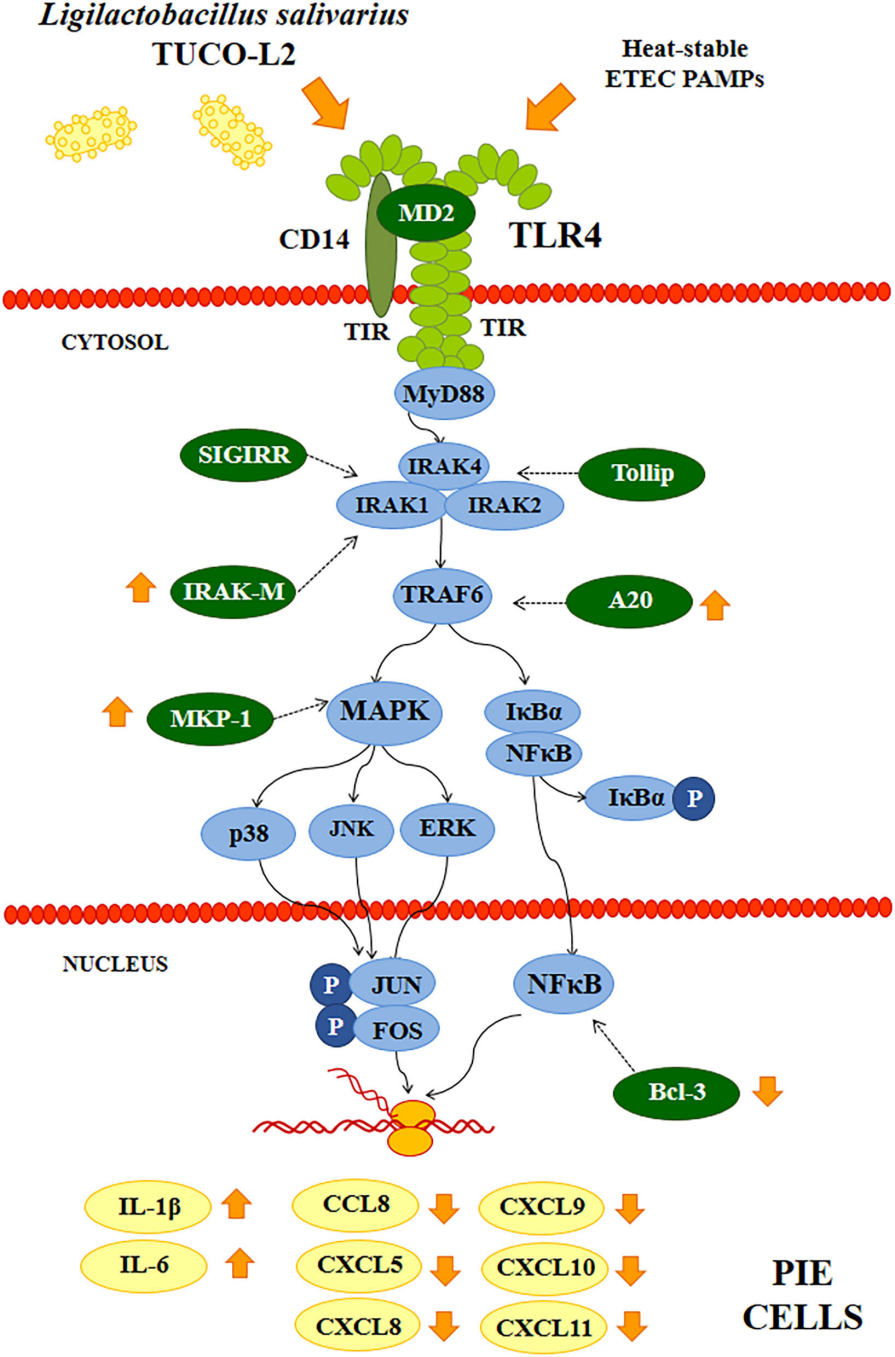
Proposed mechanism of the immunomodulatory activity of *Ligilactobacillus salivarius* TUCO-L2 isolated from llama (*Lama glama*) milk in porcine intestinal epithelial (PIE) cells. PIE cells were stimulated with the TUCO-L2 strain and then challenged with pathogen-associated molecular patterns (PAMPs) from enterotoxigenic *Escherichia coli* to induce the activation of Toll-like receptor (TLR)-4 resulting in differential expression of inflammatory cytokines and chemokines compared to that in ETEC PAMPs-challenged control cells. The immunomodulatory effect of *L. salivarius* TUCO-L2 is mediated by the modulation of the negative regulators of the TLR signaling pathway.

The levels of the blood and intestinal cytokines were evaluated after the challenge with *Salmonella* in untreated and TUCO-L2-treated mice. The challenge with the intestinal pathogen significantly increased the levels of all tested cytokines in the intestinal tract (Figure 14) and blood (Supplementary Figure 4). The intestinal levels of TNF-α, IL-1β, and IL-6 were increased in both groups after the challenge with *Salmonella*; however, the mice treated with *L. salivarius* TUCO-L2 had significantly lower levels of TNF-α and higher levels of IL-1β and IL-6 compared to those in the control animals (Figure 14). Similar results were obtained when the blood levels of TNF-α, IL-1β, and IL-6 were assayed (Supplementary Figure 4). The treatment of mice with the TUCO-L2 strain increased the levels of IFN-γ and IL-10 in the intestinal tract (Figure 15) and blood (Supplementary Figure 4) compared to those in the control groups after the challenge with *Salmonella*.

## Discussion

To the best of our knowledge, this is the first report describing the isolation of LAB from the milk of *Lama glama*. We showed that llama milk contains a population of LAB, including lactobacilli and cocci, similar to the milk of other camelids (Khedid et al., 2009; Dong et al., 2012; Rahmeh et al., 2019; Zhao et al., 2020). Studies of cultivable LAB from raw dromedary milk demonstrated the presence of *L. lactis* (17.5%), *S. thermophilus* (9.2%), and lactobacilli (20.8%) (Khedid et al., 2009) or *E. faecium* (20.7%), *L. lactis* (17.2%), and lactobacilli (12%) (Rahmeh et al., 2019). Similarly, we observed predominance of Gram-positive cocci in the bacterial isolates from llama milk samples with a minor population of lactobacilli (data not shown). Additional studies of the microbial composition of llama milk in a greater number of samples using a combination of culture-dependent and culture-independent techniques will enable more accurate characterization of the microbial populations present in this particular ecological niche. Although the results of the present study cannot be used to draw definitive conclusions about the microbiota of llama milk, we were able to isolate strains with considerable probiotic potential. One of the isolated strains, *L. salivarius* TUCO-L2, demonstrated several characteristics that would allow the use of this strain as a probiotic against intestinal infections. Our *in vitro, in vivo*, and *in silico* results indicate that *L. salivarius* TUCO-L2 can (a) resist adverse gastrointestinal conditions, (b) adhere to the intestinal mucosa, (c) antagonize intestinal bacterial pathogens, and (d) modulate the intestinal immune response.

a) *L. salivarius* TUCO-L2 resists adverse gastrointestinal conditions. Probiotic strains should be able to overcome extremely low pH of the gastric juice and the detergent effect of the bile salts to reach the site of action in a viable physiological state. Our functional experiments demonstrated that *L. salivarius* TUCO-L2 has these characteristics. Moreover, bioinformatics investigation detected two choloylglycine hydrolase genes in the genome of the TUCO-L2 strain, including *cbh1* that was not detected in the genomes of *L. salivarius* strains isolated from human milk. Interestingly, *L. salivarius* strains isolated from the intestinal tract of humans and chickens have one or two choloylglycine hydrolase genes, while most of the isolates from the porcine intestinal mucosa have three choloylglycine hydrolase genes (Lee et al., 2017). This difference has been associated with required higher resistance of the porcine strains to the toxic effect of bile salts since it was demonstrated that pigs secrete more bile per day compared to humans (Boyer, 2013). Our results indicate that the TUCO-L2 strain has higher similarity to the human and chicken intestinal strains than to the porcine strains with regard to its genetic resistance to bile salts. Our results suggest that the TUCO-L2 strain should be able to have probiotic effects after *in vivo* administration due to the presence of the choloylglycine hydrolase genes and ability to resist bile salts. This hypothesis is supported by a recent study that demonstrated that oral administration of *L. salivarius* FXJCJ7-2, HN26-4, or NT4-8 in mice protected against intestinal inflammatory damage induce by LPS administration (Zhai et al., 2020). These three *L. salivarius* strains improved the barrier function of the gut, reduced the intestinal histological alterations, and differentially modulated the Treg/Th17 balance in mice; however, the FXJCJ7-2 strain was more efficient in inducing the protective effect against LPS challenge. Notably, genomic comparison of the FXJCJ7-2, HN26-4, and NT4-8 strains demonstrated that *L. salivarius* FXJCJ7-2 has 19 unique genes that are not shared with the NT4-8 and HN26-4 strains. Among these genes, researchers detected several genes associated to the resistance to gastrointestinal tract conditions including those related to selenocysteine synthesis and transportation as well as to the production of the osmolyte proline (Zhai et al., 2020), which have been associated to the protection against bile salt actions and environmental stress (Wang et al., 2015).

Genomic comparison of the TUCO-L2 strain with *L. salivarius* from various host types and niches indicated that the strain from llama milk has genes that are not detected in the genomes of bacteria from human, porcine, or chicken origin. Unique genes of the TUCO-L2 strain include the genes encoding for the iron-sulfur cluster assembly and high affinity phosphate transporter and control of the PHO regulon. The PHO regulon is involved in the uptake of inorganic phosphate and is controlled by a two-component signal transduction system. Studies in *E. coli* demonstrated that this regulon is important for phosphate uptake and influences the genes involved in stress responses (Santos-Beneit, 2015). Studies in *L. casei* BL23 showed that the inactivation of the two-component signal transduction system of the PHO regulon results in a decrease in growth at pH 3 (Alcántara et al., 2011). On the other hand, LAB are presumed to be a rare bacterial group that has no iron requirement. However, the sequencing of the complete genomes of several LAB demonstrated that certain species harbor genes encoding for the components involved in iron transport (Makarova et al., 2006). The iron-sulfur cluster system of *E. coli* is activated under oxidative stress, i.e., in the presence of H_2_O_2_ (Outten et al., 2004). The sensitivity of the iron-sulfur cluster system to oxygen allows the system to act as an environmental sensor that conditions the responses of the bacteria. This system was suggested to be an important mechanism of resistance to oxidative stress in lactobacilli (Martinez et al., 2008). The ability to sense and respond to extracellular stresses is essential for bacterial survival under adverse conditions, such as low pH or high H_2_O_2_ concentrations; hence, the presence of these gene clusters in *L. salivarius* TUCO-L2 may be advantageous because of enhanced colonization of the mucosal surfaces or resistance to certain biotechnological processes used during food manufacturing.

b) *L. salivarius* TUCO-L2 adheres to the intestinal mucosa. Colonization of the gastrointestinal tract of the host is considered one of the most important properties of probiotic lactobacilli. In the present study, the results obtained using a porcine model host demonstrated that *L. salivarius* TUCO-L2 can adhere to porcine mucins and PIE cells in a manner similar to that of *L. salivarius* strains originally isolated from the porcine gastrointestinal tract (Masumizu et al., 2019). Furthermore, the genomic analysis of the TUCO-L2 strain identified two types of molecules that may be involved in adhesion: MucBP and srr adhesins. MucBPs were detected in LAB colonizing the gastrointestinal tract (Latousakis and Juge, 2018). These proteins contain an N-terminal secretion signal peptide YSIRK and a C-terminal LPxTG anchoring motif. Variable number of mub repeats in the center of the molecule mediate the binding to mucin glycans due to interactions with terminal sialic acid (Etzold et al., 2014; Gunning et al., 2016). MucBP are shaped as fiber-like structures of variable length that form appendices similar to pili (Etzold and Juge, 2014). The genomes of *Lactobacillus* species can harbor one or more types of MucBPs (Latousakis and Juge, 2018). Up to seven different MucBP orthologs were detected in the pangenome of *L. salivarius* (Lee et al., 2017). The study analyzed the genomes of *L. salivarius* strains isolated from the intestine of human, pigs, and chickens and identified a common MucBP (designated here as MucBP1) in all strains independently of the host origin. The results of the present study indicate that the genome of the *L. salivarius* strain isolated from the llama milk also contains this common MucBP1. Additionally, *L. salivarius* TUCO-L2 has a second MucBP of approximately 2,500 amino acids (designated here as MucBP2) that was also detected in the genomes of the LPM01, UCC118, DJ-sa-01, JCM1046, and ZLS006 strains.

On the other hand, the SecA2-SecY2 secretion system is associated with adherence of certain lactobacilli strains to mucosal tissues (Frese et al., 2013; De Boeck et al., 2020). This system facilitates the glycosylation of srr proteins and export of glycosylated adhesins that are involved in adhesion to the surface of the host cells (Feltcher and Braunstein, 2012; Bensing et al., 2014; Wegmann et al., 2015). Previous studies identified the genes encoding for the SecA2-SecY2 system in *L. salivarius* strains isolated from pig and chicken intestines (Lee et al., 2017). However, the analysis of the associated srr proteins did not detect any known adhesin-associated binding domain. In the present study, two srr proteins associated with the SecA2-SecY2 system of *L. salivarius* TUCO-L2 were incompletely characterized due to limitations in contig fragmentation.

Most bacteria with the SecA2-SecY2 systems produce a unique srr glycoprotein (Frese et al., 2013; De Boeck et al., 2020). However, certain Gram-positive bacteria can produce two or three srr proteins associated with the SecA2-SecY2 system. Human commensal bacterium *Streptococcus salivarius* JIM8777, which is an efficient colonizer of the oral and intestinal mucosa, expresses three non-homologous glycosylated surface proteins that have characteristics of srr adhesins and are secreted through the accessory SecA2-SecY2 system (Couvigny et al., 2017, 2018). Electron microscopy detected fibril-like structures partially covering the surface of JIM8777, which were eliminated from the bacterial surface in the *secA2* mutants. Moreover, the adhesive abilities of *S. salivarius* were partly related to the presence of cell wall-associated fibril-like structures since the *secA2* mutants had less efficient adhesion to epithelial cells or proteins of the extracellular matrix (Couvigny et al., 2017, 2018). The srr proteins mediate several interactions of *S. salivarius* with the environment and have thus emerged as the crucial host attachment factors involved in the colonization of mucosal surfaces by this commensal bacterium. Electron microscopy analysis of the TUCO-L2 strain demonstrated the presence of fibrin-like structures on bacterial surface; however, the density of the structures was significantly lower than that reported in *S. salivarius* (Couvigny et al., 2017, 2018). It is tempting to speculate that two srr proteins may have a similar function in *L. salivarius* TUCO-L2 that enables colonization of the bacteria in various mucosal tissues in llamas. Additional studies evaluating the functional properties of the putative adhesins srr1 and srr2 in the TUCO-L2 strain in the context of adhesion to mucins and PIE cells may be able to determine whether these proteins are involved in the adhesion of the *L. salivarius* strain isolated from the llama milk.

c) *L. salivarius* TUCO-L2 antagonizes intestinal bacterial pathogens. The antimicrobial effects against the major gastric and enteric bacterial pathogens are one of the most desirable properties for a probiotic strain (Liévin-Le Moal and Servin, 2014). Investigation of the antibacterial activities of *L. salivarius* TUCO-L2 demonstrated that this strain inhibits the growth of pathogenic Gram-negative bacteria of human and porcine origin. The antimicrobial activities of LAB have been associated with several molecules, including lactic acid and bacteriocin or non-ribosomal peptides (Liévin-Le Moal and Servin, 2014). Neutralization of the TUCO-L2 supernatant eliminated lactic acid responsible for pathogen inhibition. Thus, *in silico* analysis was performed by using Bagel 4, blastp, and the gene annotation RAST platform to identify antibacterial substances produced by the TUCO-L2 strain. *In silico* analysis detected the presence of bacteriocin-related immunity genes possibly related to a prepeptide or a synthesis-inducing factor. Interestingly, we were unable to detect similarities of these molecules with any know bacteriocin by blastp (peptide sequences) or Bagel 4 (gene sequences) comparison. This result suggests the presence of a new bacteriocin synthetized by *L. salivarius* TUCO-L2 strain, which has an anti-Gram-negative effect. These findings strongly encourage further studies aimed to purify, sequence, and characterize this bacteriocin.

Probiotics strains with good adhesion to intestinal epithelial cells and mucins can block the adherence of the pathogens by competition for the host cell binding sites (Monteagudo-Mera et al., 2019). This transient colonization of probiotic bacteria can lead to competitive exclusion of the pathogens. Adhesion includes attachment of the bacterial cells to the host cells and to other bacterial cells of different species (coaggregation) or same species (autoaggregation). Moreover, multiple studies have shown that a potential protective role of probiotic LAB is mediated by binding of the pathogens into coaggregates thus inhibiting the biofilm formation that is frequently involved in infections (reviewed in Monteagudo-Mera et al., 2019). The results of this study indicate that *L. salivarius* TUCO-L2 binds intestinal epithelial cells and mucins and is capable of autoaggregation and coaggregation with intestinal pathogens. These data suggest that this strain can potentially promote competitive exclusion and prevent the biofilm formation by the pathogens in the intestinal mucosa.

The colonization of the intestinal surface and production of a bacteriocin by *L. salivarius* TUCO-L2 may help to avoid the colonization by enteropathogens. These antimicrobial effects of probiotic bacteria are unlikely to have a deleterious effect on the commensal intestinal bacteria (reviewed in Monteagudo-Mera et al., 2019). Our results confirm this assertion since we did not detect any signs of intestinal dysbiosis when the TUCO-L2 strain was administered to mice.

d) *L. salivarius* TUCO-L2 modulates the intestinal immune response. In addition to adherence to PIE cells, our data indicate that *L. salivarius* TUCO-L2 differentially modulates the innate immune response triggered by TLR4 activation in this cell line (Figure 15). We have previously used the PIE cell line to study the influence of immunomodulatory probiotic (immunobiotic) lactobacilli on the TLR4-mediated immune response induced by ETEC PAMPs. We demonstrated that the challenge of PIE cells with ETEC PAMPs activates the MAPK and NF-kB signaling pathways leading to the expression of inflammatory cytokines, including *IL-1*β and *IL-6* (Shimazu et al., 2012; Wachi et al., 2014; Garcia-Castillo et al., 2019), and several chemokines, including *CCL8, CXCL5, CXCL8, CXCL9, CXCL10*, and *CXCL11* (Kobayashi et al., 2016). Then, these cytokines and chemokines were assayed to evaluate the potential immunomodulatory properties of the TUCO-L2 strain from llama milk. *L. salivarius* TUCO-L2 increased the expression of *IL-1*β and *IL-6* and reduced the expression of all tested chemokines compared to those in the control cells (Figure 15). Previously, we demonstrated that prestimulation of PIE cells with the immunobiotic strain *Lactobacillus jensenii* TL2937 differentially modulated the expression of inflammatory factors produced in response to ETEC PAMPs challenge. The TL2937 strain increased the expression of *IL-1*β and significantly reduced the expression levels of *IL-6, CCL8, CXCL5, CXCL8, CXCL9, CXCL10*, and *CXCL11* (Kobayashi et al., 2016). This strong anti-inflammatory effect was related to the upregulation of the negative regulators *MKP-1, A20*, and *Bcl-3* in PIE cells (Shimazu et al., 2012). Moreover, *L. jensenii* TL2937 efficiently reduced intestinal inflammation associated with weaning in piglets (Suda et al., 2014). We also reported that immunobiotic *Limosilactobacillus fermentum* UCO-979c (basonym: *Lactobacillus fermentum* UCO-979c) significantly reduced the levels of *CXCL8, CXCL9, CXCL10*, and *CXCL11* and increased the expression levels of *IL-6* and *CCL8* in PIE cells challenged with ETEC PAMPs (Garcia-Castillo et al., 2019). This mixed stimulant/anti-inflammatory effect induced by *L. fermentum* UCO-979c was associated with a reduction in the expression of *Tollip* and *MKP-1* and upregulation of *SIGIRR* and *Bcl-3* in ETEC PAPMs-challenged PIE cells. The UCO-979c strain was able to protect against intestinal pathogens *in vitro* and *in vivo* (Garcia-Castillo et al., 2019). The results of this work indicated that *L. salivarius* TUCO-L2 has a mixed stimulant/anti-inflammatory effect similar to the UCO-979c strain. In fact, the analysis of the expression of the negative regulators of the TRL4 signaling pathway in PIE cells indicated that the TUCO-L2 strain enhances the expression of *IRAK-M, MKP-1*, and *A20* and reduces the expression of *Bcl-3* in PIE cells. Moreover, *in vivo* experiments in mice clearly demonstrated that the TUCO-L2 strain increases the resistance to *Salmonella* infection by differentially modulating the immune response. *L. salivarius* TUCO-L2 demonstrated a mixed stimulant/anti-inflammatory effect *in vivo* because it reduced the levels of TNF-α and increased the levels of IL-1β, IL-6, IFN-γ, and IL-10 in the intestine and blood of treated animals compared to those in the control mice.

The intestinal mucosa needs to maintain balanced TLR activation to protect against invading pathogens and avoid uncontrolled inflammatory responses that can lead to tissue damage (Allaire et al., 2018). Appropriate balance of inflammatory cytokines and chemokines is necessary to efficiently manifest this function. The changes in the cytokine profiles induced by *L. salivarius* TUCO-L2 in PIE cells after the stimulation with ETEC PAPMs and in mice after the challenge with *Salmonella* indicate that this strain positively influences the intestinal immune response triggered by Gram-negative pathogens. On the one hand, reduced chemokines expression and increased levels of the immunoregulatory cytokine IL-10 may account for the effects of the TUCO-L2 strain on the regulation of the activation and recruitment of inflammatory cells, such as neutrophils, thus limiting the inflammatory damage in the intestinal mucosa. On the other hand, an increase in IL-1β and IL-6 may help to eliminate the pathogens by modulating the effector functions of T cells (Siegmund et al., 2001; Kuhn et al., 2018). Activation of the inflammasome in intestinal epithelial cells is involved in the production of the active form of IL-1β, which collaborate in the activation and differentiation of T cells that produce IL-17 or IFN-γ. Additionally, IL-6 in the gut promotes T cell expansion and stimulates Th1 cell-mediated inflammation. Then, it is tempting to speculate that differential expression of IL-1β and IL-6 induced by the TUCO-L2 strain in intestinal epithelial cells favors efficient development of functional Th1. This hypothesis is in agreement with an increase in the levels of IFN-γ detected in mice treated with *L. salivarius* TUCO-L2 and challenged with *Salmonella*. Additional studies are needed to characterize the influence of the TUCO-L2 strain on intestinal immunity, including qualitative and quantitative evaluation of antigen-presenting cells, effector T cells, and Treg cells.

In addition to the effect on intestinal immune cell populations, it would be of interest to identify bacterial molecules of the TUCO-L2 strain that are involved in molecular interaction with the immune system. The results of electron microscopy analysis in the present study demonstrated that *L. salivarius* TUCO-L2 can produce EPS. Genomic analysis was used to characterize EPS of the llama milk strain. Genes of EPS cluster 1 were not detected in the genome of *L. salivarius* TUCO-L2. This result is in agreement with previous genomic studies of Harris et al. (2017) that demonstrated that EPS cluster 1 is mainly detected in *L. salivarius* strains of human origin suggesting that this gene cluster encodes a trait involved in adaptation to the human gastrointestinal tract. All *L. salivarius* strains investigated in the present study, including the TUCO-L2 strain, contained genes of EPS clusters 2 and 3; however, evaluation of the phylogenetic clustering based in the nucleotide sequences of 12 conserved genes indicated a certain divergence. These results are in agreement with previous genomics studies of Lee et al. (2017) that used 24 conserved EPS genes to evaluate the differences between *L. salivarius* isolated from humans, pigs, and chickens and that detected divergence associated with the hosts. The study suggested that the point mutations in the EPS genes rather than gene acquisition/loss may explain the production of different EPS by *L. salivarius* strains. Moreover, Harris et al. (2017) demonstrated variable abundance of the genes of glycosyltransferase families in 42 *L. salivarius* strains emphasizing that strain-specific sets of glycosyltransferases may be involved in the generation of variable structures of EPS associated with different interaction with abiotic and biotic environmental factors. Previous findings and genomic analysis performed in the present study suggest that *L. salivarius* TUCO-L2 may produce an EPS with unique characteristics. Further characterization of the TUCO-L2 EPS may be able to determine its role in the probiotic or immunobiotic properties of this strain considering that EPS of some lactobacilli are the key molecules associated with bacterial adhesion (Živković et al., 2016) or immunomodulatory effects (Wachi et al., 2014; Kanmani et al., 2018a,b).

In conclusion, this is the first report on the isolation and characterization of a potential probiotic/immunobiotic strain from llama milk. *In vitro* functional studies complemented with genomic analysis and *in vivo* studies in mice demonstrated that *L. salivarius* TUCO-L2 has several characteristics of probiotic strains including resistance to pH and high bile salt concentrations, antimicrobial activity against Gram-negative intestinal pathogens, and adherence to mucins and epithelial cells. Moreover, similar to other immunobiotic strains, *L. salivarius* TUCO-L2 differentially modulates the innate immune response triggered by TLR4 activation and improves the protection against Gram-negative intestinal pathogens. Thus, this study provides access to several directions of investigations to expand the characterization of the TUCO-L2 strain and position it as a probiotic or immunobiotic strain that can be used against infections in humans or animals, such as llamas.

## Data Availability Statement

The datasets generated for this study can be found in online repositories. The names of the repository/repositories and accession number(s) can be found at: https://www.ncbi.nlm.nih.gov/genbank/, SOPE01000000.

## Ethics Statement

The animal study was reviewed and approved by The CERELA Institutional Animal Care and Use Committee (San Miguel de Tucuman, Tucuman, Argentina).

## Author Contributions

SQ-V, YS, HK, and JV designed the study. SQ-V, FM, LAr, BZ, and MT performed *in vitro* studies. LAl, IA, and LAr, performed the bioinformatics studies. LAr and JV performed *in vivo* studies. SQ-V, YS, LAr, and LAl performed the statistical analysis. SQ-V, HK, and JV analyzed the data. HK and JV wrote the manuscript. All authors read and approved the manuscript.

## Conflict of Interest

The authors declare that the research was conducted in the absence of any commercial or financial relationships that could be construed as a potential conflict of interest.

## Supplementary Material

Supplementary Figure 1 Microbiological characterization of *Ligilactobacillus salivarius* TUCO-L2. The TUCO-L2 strain is a short rod-shaped Gram-positive bacterium that aggregates in MRS broth and grows as white pearl-like colonies in MRS agar.

Supplementary Figure 2 Genomic comparison of the pilus operon of *Ligilactobacillus salivarius* TUCO-L2 isolated from llama (*Lama glama*) milk with that of the strains of the same species. The pilus operon genes in the genomes of *L. salivarius* JCM1047, DSM20555, ATCC11741, gul1, gul2, and A3iob were identified by search of the genome of the TUCO-L2 strain.

Supplementary Figure 3 Genomic comparison of the SeA2-SecY2 accessory secretion system of *Ligilactobacillus salivarius* TUCO-L2 isolated from llama (*Lama glama*) milk with the gene clusters of the *L. salivarius* strains from the intestinal tract of pig (JCM1046) and chicken (DJ-sa-01). Gene organization of the SeA2-SecY2 accessory secretion system of selected strains is shown. Conserved glycosyltransferases are shown in orange, and non-conserved glycosyltransferases are shown in light green. Putative srr adhesins are shown in dark green. Gray connections indicate high homology between the genes.

Supplementary Figure 4 The effect of *Ligilactobacillus salivarius* TUCO-L2 isolated from llama (*Lama glama*) milk on the systemic immune response of adult mice to *Salmonella* infection. *L. salivarius* TUCO-L2 was administered to male 6-week-old Balb/c mice for 5 consecutive days at a dose of 10^8^ cells/mouse/day in drinking water. Untreated mice were used as a control. TUCO-L2-treated and control mice were challenged by oral administration of 10^7^ cells/mouse of *S. typhimurium* (20LD50) on day 6. Mice were sacrificed on day 2 after the infection, and the levels of serum TNF-α, IFN-γ, IL-1β, IL-6, and IL-10 were measured by ELISA. Untreated and uninfected mice were used for comparison (basal group). Each parameter was assayed in 5–6 mice per group. Letters indicate significant differences (*P* < 0.05), a < b < c.

Supplementary Table 1 Evaluation of general probiotic properties. Resistance to NaCl and Oxgall.

Supplementary Table 2 General genomic features of *Ligilactobacillus salivarius* strains used in this work.

Supplementary Table 3 List of unique genes found in the genome of *Ligilactobacillus salivarius* TUCO-L2 compared with the strains isolated from human milk *L. salivarius* CECT 5713 and LPM01.

Supplementary Table 4 List of unique genes found in the genome of *Ligilactobacillus salivarius* TUCO-L2 compared with the strains isolated from human intestine *L. salivarius* UCC118 and REN.

Supplementary Table 5 List of unique genes found in the genome of *Ligilactobacillus salivarius* TUCO-L2 compared with the strains isolated from porcine intestine *L. salivarius* JCM1046 and ZLS006.

Supplementary Table 6 List of unique genes found in the genome of *Ligilactobacillus salivarius* TUCO-L2 compared with the strains isolated from chicken intestine *L. salivarius* DJ-sa-01 and CICC23174.

Supplementary Table 7 BAGEL 4 and blastp analysis for the search of bacteriocins in the genome of Ligilactobacillus salivarius TUCO-L2.
