# Supplementary figures and images for: Functional and Genomic Characterization of Ligilactobacillus salivarius TUCO-L2 Isolated From Lama glama Milk: A Promising Immunobiotic Strain to Combat Infections
Source: Front Microbiol. 2020 Dec 8;11:608752. doi: 10.3389/fmicb.2020.608752 (PMC7752859; doi:10.3389/fmicb.2020.608752)

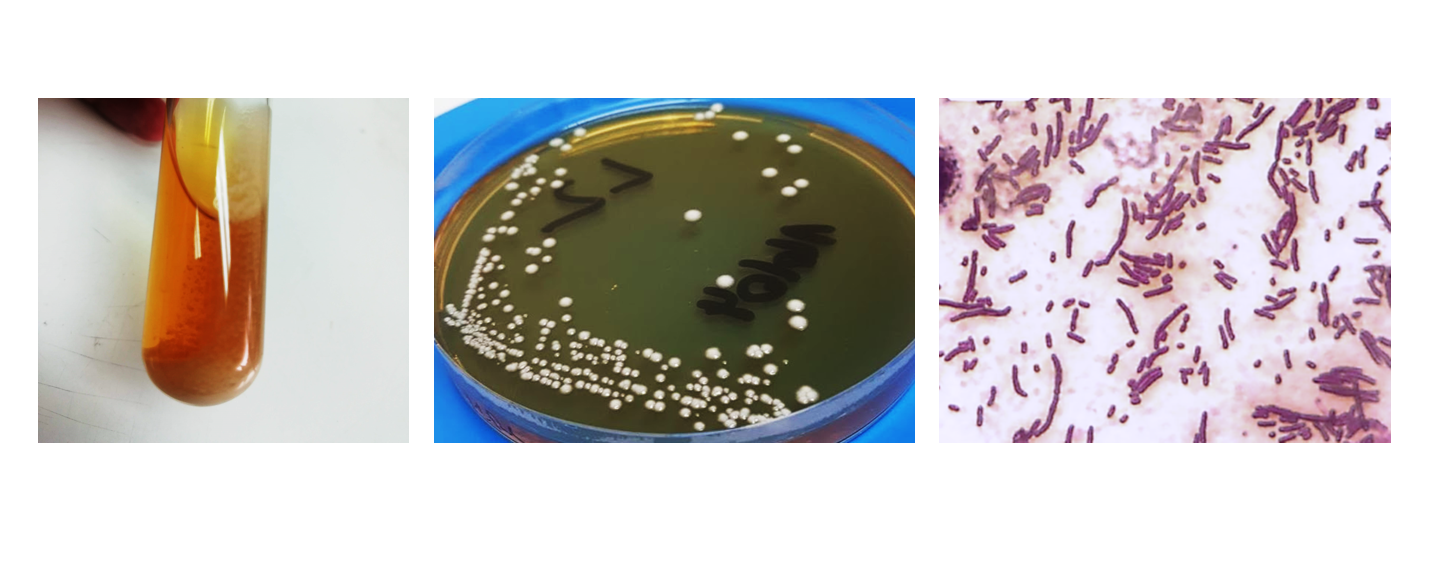

Supplement: Supplementary Figure 1 — Microbiological characterization of Ligilactobacillus salivarius TUCO-L2. The TUCO-L2 strain is a short rod-shaped Gram-positive bacterium that aggregates in MRS broth and grows as white pearl-like colonies in MRS agar. [file Image_1.TIF]

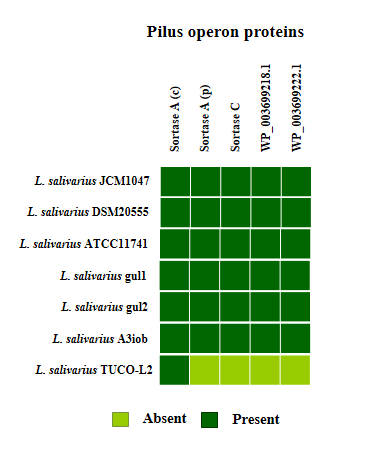

Supplement: Supplementary Figure 2 — Genomic comparison of the pilus operon of Ligilactobacillus salivarius TUCO-L2 isolated from llama (Lama glama) milk with that of the strains of the same species. The pilus operon genes in the genomes of L. salivarius JCM1047, DSM20555, ATCC11741, gul1, gul2, and A3iob were identified by search of the genome of the TUCO-L2 strain. [file Image_2.TIF]

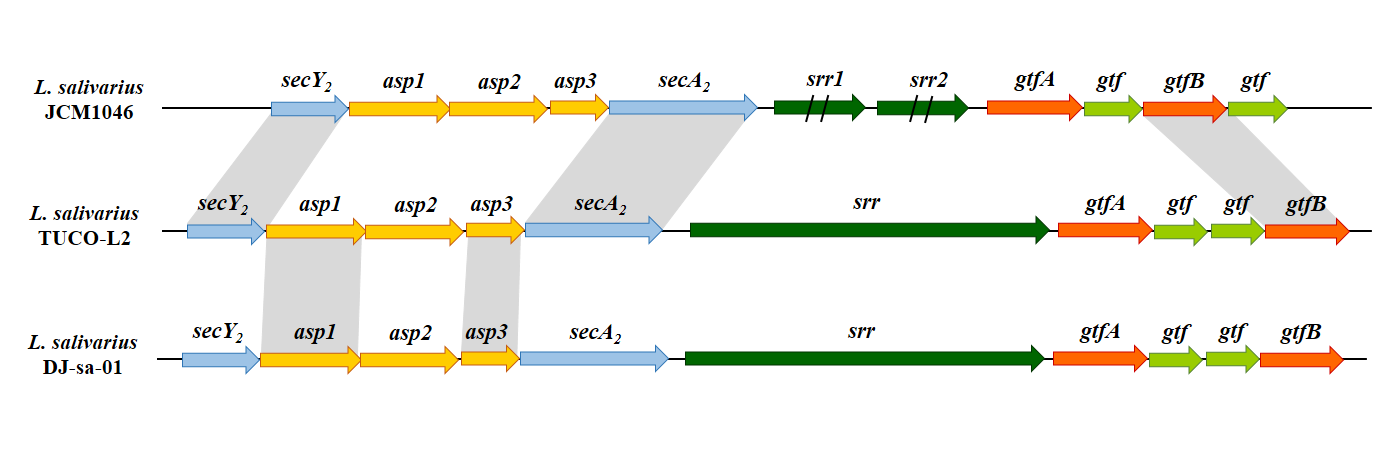

Supplement: Supplementary Figure 3 — Genomic comparison of the SeA2-SecY2 accessory secretion system of Ligilactobacillus salivarius TUCO-L2 isolated from llama (Lama glama) milk with the gene clusters of the L. salivarius strains from the intestinal tract of pig (JCM1046) and chicken (DJ-sa-01). Gene organization of the SeA2-SecY2 accessory secretion system of selected strains is shown. Conserved glycosyltransferases are shown in orange, and non-conserved glycosyltransferases are shown in light green. Putative srr adhesins are shown in dark green. Gray connections indicate high homology between the genes. [file Image_3.TIF]

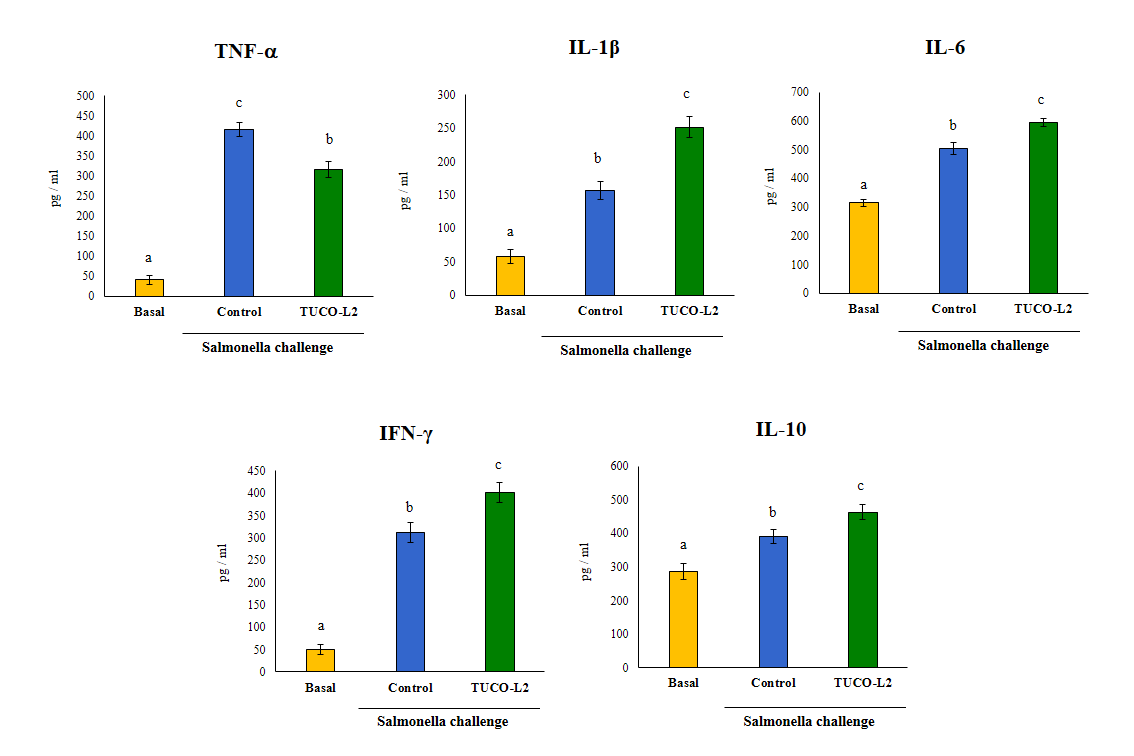

Supplement: Supplementary Figure 4 — The effect of Ligilactobacillus salivarius TUCO-L2 isolated from llama (Lama glama) milk on the systemic immune response of adult mice to Salmonella infection. L. salivarius TUCO-L2 was administered to male 6-week-old Balb/c mice for 5 consecutive days at a dose of 108 cells/mouse/day in drinking water. Untreated mice were used as a control. TUCO-L2-treated and control mice were challenged by oral administration of 107 cells/mouse of S. typhimurium (20LD50) on day 6. Mice were sacrificed on day 2 after the infection, and the levels of serum TNF-α, IFN-γ, IL-1β, IL-6, and IL-10 were measured by ELISA. Untreated and uninfected mice were used for comparison (basal group). Each parameter was assayed in 5–6 mice per group. Letters indicate significant differences (P < 0.05), a < b < c. [file Image_4.TIF]
