# Supplementary material for: Functional and Genomic Characterization of Ligilactobacillus salivarius TUCO-L2 Isolated From Lama glama Milk: A Promising Immunobiotic Strain to Combat Infections
Source: Front Microbiol. 2020 Dec 8;11:608752. doi: 10.3389/fmicb.2020.608752 (PMC7752859; doi:10.3389/fmicb.2020.608752)
Supplement: Supplementary Table 1 — Evaluation of general probiotic properties. Resistance to NaCl and Oxgall. [file Table_1.docx]

**Supplementary Table 1.** Evaluation of general probiotic properties. Resistance to NaCl and Oxgall.

| *Lama glama* strains | TUCO-L1 | TUCO-L2 | TUCO-L3 | TUCO-L5 |
| --- | --- | --- | --- | --- |
| % NaCl w/v | | | | |
| 2 | Resistant | Resistant | Resistant | Resistant |
| 6 | Resistant | Resistant | Susceptible | Susceptible |
| 9 | Resistant | Resistant | Susceptible | Susceptible |
| % Oxgall w/v | | | | |
| 0.5 | Resistant | Resistant | Resistant | Resistant |
| 2 | Resistant | Resistant | Resistant | Resistant |
| 5 | Resistant | Resistant | Resistant | Resistant |
