# Supplementary material for: Functional and Genomic Characterization of Ligilactobacillus salivarius TUCO-L2 Isolated From Lama glama Milk: A Promising Immunobiotic Strain to Combat Infections
Source: Front Microbiol. 2020 Dec 8;11:608752. doi: 10.3389/fmicb.2020.608752 (PMC7752859; doi:10.3389/fmicb.2020.608752)
Supplement: Supplementary Table 2 — General genomic features of Ligilactobacillus salivarius strains used in this work. [file Table_2.docx]

| ***Ligilactobacillus salivarius* strain** | **Host** | **Sample** | **Genome size**  **(bp)** | **G+C content (%)** | **Protein-coding genes** | **NCBI Reference Sequence** |
| --- | --- | --- | --- | --- | --- | --- |
| TUCO-L2 | *Llama glama* | Milk | 1,600,747 | 33.0 | 1,691 | SOPE01000000 |
| LPM01 | *Homo sapiens* | Milk | 1,788,723 | 33.0 | 1,717 | NZ_LT604074.1 |
| CECT 5713 | *Homo sapiens* | Milk | 1,828,169 | 32.9 | 1,884 | NZ_CP017107.1 |
| JCM1046 | *Sus scrofa* | Intestine | 1,836,297 | 33.1 | 1,803 | NZ_CP007646.1 |
| ZLS006 | *Sus scrofa* | Intestine | 2,177,581 | 33.2 | 2,114 | NZ_CP020858.1 |
| CICC 23174 | *Gallus gallus* | Intestine | 1,746,897 | 33.0 | 1,606 | CP002034.1 |
| DJ-sa-01 | *Gallus gallus* | Intestine | 1,870,629 | 33.0 | 1,719 | NZ_CP029616.1 |
| UCC118 | *Homo sapiens* | Intestine | 1,827,111 | 32.9 | 1,807 | NC_006529.1 |
| REN | *Homo sapiens* | Intestine | 1,928,516 | 32.9 | 1,861 | NZ_CP011403.1 |

**Supplementary Table 2.** General genomic features of *Ligilactobacillus salivarius* strains used in this work.
