# Supplementary material for: Functional and Genomic Characterization of Ligilactobacillus salivarius TUCO-L2 Isolated From Lama glama Milk: A Promising Immunobiotic Strain to Combat Infections
Source: Front Microbiol. 2020 Dec 8;11:608752. doi: 10.3389/fmicb.2020.608752 (PMC7752859; doi:10.3389/fmicb.2020.608752)
Supplement: Supplementary Table 5 — List of unique genes found in the genome of Ligilactobacillus salivarius TUCO-L2 compared with the strains isolated from porcine intestine L. salivarius JCM1046 and ZLS006. [file Table_5.docx]

**Supplementary Table 5.** List of unique genes found in the genome of *Ligilactobacillus salivarius* TUCO-L2 compared with the strains isolated from porcine intestine *L. salivarius* JCM1046 and ZLS006.

| **Gene** | **Annotation** | **TUCO-L2 ID** |
| --- | --- | --- |
| *asp2* | Accessory Sec system protein Asp2 | SOPE01000100.1_00759 |
| *casC* | CRISPR system Cascade subunit CasC | SOPE01000100.1_00947 |
| *ygbT* | CRISPR-associated endonuclease Cas1 | SOPE01000100.1_00950 |
| *bglH_1* | Aryl-phospho-beta-D-glucosidase BglH | SOPE01000100.1_00886 |
| *bglH_2* | Aryl-phospho-beta-D-glucosidase BglH | SOPE01000100.1_00995 |
| *celD* | PTS system cellobiose-specific EIIA component | SOPE01000100.1_00793 |
| *csbB_1* | Putative glycosyltransferase CsbB | SOPE01000100.1_00536 |
| *epsE_1* | Putative glycosyltransferase EpsE | SOPE01000100.1_00264 |
| *epsE_2* | Putative glycosyltransferase EpsE | SOPE01000100.1_01201 |
| *cap8A* | Capsular polysaccharide type 8 biosynthesis protein cap8A | SOPE01000100.1_00096 |
| *epsJ_1* | putative glycosyltransferase EpsJ | SOPE01000100.1_00104 |
| *epsJ_2* | putative glycosyltransferase EpsJ | SOPE01000100.1_00112 |
| *wbbI_2* | Beta-1,6-galactofuranosyltransferase WbbI | SOPE01000100.1_00811 |
| *epsJ_3* | putative glycosyltransferase EpsJ | SOPE01000100.1_00812 |
| *epsJ_4* | putative glycosyltransferase EpsJ | SOPE01000100.1_00813 |
| *wbbI_3* | Beta-1,6-galactofuranosyltransferase WbbI | SOPE01000100.1_01116 |
| *epsJ_5* | putative glycosyltransferase EpsJ | SOPE01000100.1_01199 |
| *gtfC* | Glucosyltransferase-SI | SOPE01000100.1_01094 |
| *tagE* | Poly(glycerol-phosphate) alpha-glucosyltransferase | SOPE01000100.1_01200 |
| *yjaB* | putative N-acetyltransferase YjaB | SOPE01000100.1_01372 |
| *wfgD* | UDP-Glc:alpha-D-GlcNAc-diphosphoundecaprenol beta-1,3-glucosyltransferase WfgD | SOPE01000100.1_00690 |
| *iolA* | Methylmalonate semialdehyde dehydrogenase [acylating] | SOPE01000100.1_00635 |
| *iolB* | 5-deoxy-glucuronate isomerase | SOPE01000100.1_00920 |
| *iolD* | 3D-(3,5/4)-trihydroxycyclohexane-1,2-dione hydrolase | SOPE01000100.1_00981 |
| *iolE* | Inosose dehydratase | SOPE01000100.1_01209 |
| *iolG* | Inositol 2-dehydrogenase/D-chiro-inositol 3-dehydrogenase | SOPE01000100.1_00982 |
| *iolI* | Inosose isomerase | SOPE01000100.1_01176 |
| *apbE* | FAD:protein FMN transferase | SOPE01000100.1_00799 |
| *aroD* | 3-dehydroquinate dehydratase | SOPE01000100.1_01172 |
| *aroE* | Shikimate dehydrogenase (NADP(+)) | SOPE01000100.1_01173 |
| *arsC* | Glutaredoxin arsenate reductase | SOPE01000100.1_00037 |
| *bacC_3* | Dihydroanticapsin 7-dehydrogenase | SOPE01000100.1_01175 |
| *cadA_1* | putative cadmium-transporting ATPase | SOPE01000100.1_00580 |
| *cdd* | Cytidine deaminase | SOPE01000100.1_00791 |
| *copZ* | Copper chaperone CopZ | SOPE01000100.1_00581 |
| *cynR* | HTH-type transcriptional regulator CynR | SOPE01000100.1_00387 |
| *dacA_2* | D-alanyl-D-alanine carboxypeptidase DacA | SOPE01000100.1_01019 |
| *dps* | DNA protection during starvation protein | SOPE01000100.1_00583 |
| *gerN_3* | Na(+)/H(+)-K(+) antiporter GerN | SOPE01000100.1_00795 |
| *gldA* | Glycerol dehydrogenase | SOPE01000100.1_01300 |
| *gltC_2* | HTH-type transcriptional regulator GltC | SOPE01000100.1_01177 |
| *cysM* | Cysteine synthase | SOPE01000100.1_00022 |
|  | Putative phosphatase | SOPE01000100.1_00044 |
| *rsmF* | Ribosomal RNA small subunit methyltransferase F | SOPE01000100.1_00066 |
| *paiA* | Spermidine/spermine N(1)-acetyltransferase | SOPE01000100.1_00067 |
| *ybaN* | Inner membrane protein YbaN | SOPE01000100.1_00355 |
| *yhdG_2* | putative amino acid permease YhdG | SOPE01000100.1_00567 |
| *sdhB* | L-serine dehydratase, beta chain | SOPE01000100.1_00570 |
| *toxA_1* | Toxin A | SOPE01000100.1_00599 |
| *xerC_2* | Tyrosine recombinase XerC | SOPE01000100.1_00640 |
| *xre* | HTH-type transcriptional regulator Xre | SOPE01000100.1_00644 |
| *lexA_1* | LexA repressor | SOPE01000100.1_00645 |
| *gapA1* | Glyceraldehyde-3-phosphate dehydrogenase 1 | SOPE01000100.1_00891 |
| *toxA_2* | Toxin A | SOPE01000100.1_00971 |
| *bglF_2* | PTS system beta-glucoside-specific EIIBCA component | SOPE01000100.1_00996 |
| *toxA_3* | Toxin A | SOPE01000100.1_01117 |
| *mvaA* | 3-hydroxy-3-methylglutaryl-coenzyme A reductase | SOPE01000100.1_00534 |
| *smc_4* | Chromosome partition protein Smc | SOPE01000100.1_01299 |
| *adhR_2* | HTH-type transcriptional regulator AdhR | SOPE01000100.1_01330 |
| *radD* | Putative DNA repair helicase RadD | SOPE01000100.1_01381 |
| *gspA* | General stress protein A | SOPE01000100.1_00356 |
| *hmo* | 4-hydroxymandelate oxidase | SOPE01000100.1_01152 |
| *immR_1* | HTH-type transcriptional regulator ImmR | SOPE01000100.1_00643 |
| *Int-Tn_1* | Transposase from transposon Tn916 | SOPE01000100.1_00951 |
| *licB* | Lichenan-specific phosphotransferase enzyme IIB component | SOPE01000100.1_01211 |
| *licT* | Transcription antiterminator LicT | SOPE01000100.1_00997 |
| *lyc* | Autolytic lysozyme | SOPE01000100.1_01122 |
| *nudG_2* | CTP pyrophosphohydrolase | SOPE01000100.1_00884 |
| *pepQ_2* | Xaa-Pro dipeptidase | SOPE01000100.1_00714 |
| *qacA* | Antiseptic resistance protein | SOPE01000100.1_00389 |
| *qorB_2* | Quinone oxidoreductase 2 | SOPE01000100.1_00390 |
| *rclA* | putative pyridine nucleotide-disulfide oxidoreductase RclA | SOPE01000100.1_00354 |
| *rluD_3* | Ribosomal large subunit pseudouridine synthase D | SOPE01000100.1_01326 |
| *tuaB* | Teichuronic acid biosynthesis protein TuaB | SOPE01000100.1_00360 |
| *ugd* | UDP-glucose 6-dehydrogenase | SOPE01000100.1_00676 |
| *urdA* | Urocanate reductase | SOPE01000100.1_01174 |
| *xynY* | Endo-1,4-beta-xylanase Y | SOPE01000100.1_00391 |
| *ydiM_1* | Inner membrane transport protein YdiM | SOPE01000100.1_00122 |
| *ydiM_2* | Inner membrane transport protein YdiM | SOPE01000100.1_01178 |
| *yhdG_1* | putative amino acid permease YhdG | SOPE01000100.1_00566 |
| *yhjE* | Inner membrane metabolite transport protein YhjE | SOPE01000100.1_00168 |
| *yidK* | putative symporter YidK | SOPE01000100.1_01210 |
| *ykfC* | Gamma-D-glutamyl-L-lysine endopeptidase | SOPE01000100.1_01126 |
| *yvdT_1* | putative HTH-type transcriptional regulator YvdT | SOPE01000100.1_00167 |
| *yvgN* | Glyoxal reductase | SOPE01000100.1_00388 |
