# Supplementary material for: Functional and Genomic Characterization of Ligilactobacillus salivarius TUCO-L2 Isolated From Lama glama Milk: A Promising Immunobiotic Strain to Combat Infections
Source: Front Microbiol. 2020 Dec 8;11:608752. doi: 10.3389/fmicb.2020.608752 (PMC7752859; doi:10.3389/fmicb.2020.608752)
Supplement: Supplementary Table 6 — List of unique genes found in the genome of Ligilactobacillus salivarius TUCO-L2 compared with the strains isolated from chicken intestine L. salivarius DJ-sa-01 and CICC23174. [file Table_6.docx]

**Supplementary Table 6.** List of unique genes found in the genome of *Ligilactobacillus salivarius* TUCO-L2 compared with the strains isolated from chicken intestine *L. salivarius* DJ-sa-01 and CICC23174.

| **Gene** | **Annotation** | **TUCO-L2** |
| --- | --- | --- |
| *cbh_1* | Choloylglycine hydrolase | SOPE01000100.1_00308 |
| *bglF_2* | PTS system beta-glucoside-specific EIIBCA component | SOPE01000100.1_00996 |
| *bglH_1* | Aryl-phospho-beta-D-glucosidase BglH | SOPE01000100.1_00886 |
| *bglH_2* | Aryl-phospho-beta-D-glucosidase BglH | SOPE01000100.1_00995 |
| *bglK* | Beta-glucoside kinase | SOPE01000100.1_01167 |
| *cap8A* | Capsular polysaccharide type 8 biosynthesis protein cap8A | SOPE01000100.1_00096 |
| *celD* | PTS system cellobiose-specific EIIA component | SOPE01000100.1_00793 |
| *epsE_1* | Putative glycosyltransferase EpsE | SOPE01000100.1_00264 |
| *epsE_2* | Putative glycosyltransferase EpsE | SOPE01000100.1_01201 |
| *epsJ_1* | putative glycosyltransferase EpsJ | SOPE01000100.1_00104 |
| *epsJ_2* | putative glycosyltransferase EpsJ | SOPE01000100.1_00112 |
| *epsJ_4* | putative glycosyltransferase EpsJ | SOPE01000100.1_00813 |
| *epsJ_5* | putative glycosyltransferase EpsJ | SOPE01000100.1_01199 |
| *fsaA* | Fructose-6-phosphate aldolase 1 | SOPE01000100.1_00144 |
| *epsJ_3* | putative glycosyltransferase EpsJ | SOPE01000100.1_00812 |
| *gtfC* | Glucosyltransferase-SI | SOPE01000100.1_01094 |
| *rhaA* | L-rhamnose isomerase | SOPE01000100.1_01090 |
| *rhaB* | Rhamnulokinase | SOPE01000100.1_01089 |
| *rhaD* | Rhamnulose-1-phosphate aldolase | SOPE01000100.1_00693 |
| *srlA* | PTS system glucitol/sorbitol-specific EIIC component | SOPE01000100.1_00124 |
| *srlE* | PTS system glucitol/sorbitol-specific EIIB component | SOPE01000100.1_00123 |
| *wbbI_2* | Beta-1,6-galactofuranosyltransferase WbbI | SOPE01000100.1_00811 |
| *wbbI_3* | Beta-1,6-galactofuranosyltransferase WbbI | SOPE01000100.1_01116 |
| *iolA* | Methylmalonate semialdehyde dehydrogenase | SOPE01000100.1_00635 |
| *iolB* | 5-deoxy-glucuronate isomerase | SOPE01000100.1_00920 |
| *iolD* | 3D-(3,5/4)-trihydroxycyclohexane-1,2-dione hydrolase | SOPE01000100.1_00981 |
| *iolE* | Inosose dehydratase | SOPE01000100.1_01209 |
| *iolG* | Inositol 2-dehydrogenase/D-chiro-inositol 3-dehydrogenase | SOPE01000100.1_00982 |
| *iolI* | Inosose isomerase | SOPE01000100.1_01176 |
| *adhE* | Aldehyde-alcohol dehydrogenase | SOPE01000100.1_01245 |
| *aroD* | 3-dehydroquinate dehydratase | SOPE01000100.1_01172 |
| *aroE* | Shikimate dehydrogenase (NADP(+)) | SOPE01000100.1_01173 |
| *bacC_1* | Dihydroanticapsin 7-dehydrogenase | SOPE01000100.1_00993 |
| *bacC_3* | Dihydroanticapsin 7-dehydrogenase | SOPE01000100.1_01175 |
| *cadA_1* | putative cadmium-transporting ATPase | SOPE01000100.1_00580 |
| *copZ* | Copper chaperone CopZ | SOPE01000100.1_00581 |
| *csbB_1* | Putative glycosyltransferase CsbB | SOPE01000100.1_00536 |
| *csbC* | putative metabolite transport protein CsbC | SOPE01000100.1_00266 |
| *cysM* | Cysteine synthase | SOPE01000100.1_00022 |
| *dapL* | LL-diaminopimelate aminotransferase | SOPE01000100.1_00772 |
| *desK_1* | Sensor histidine kinase DesK | SOPE01000100.1_00311 |
| *desR* | Transcriptional regulatory protein DesR | SOPE01000100.1_00310 |
| *dosC* | Diguanylate cyclase DosC | SOPE01000100.1_00849 |
| *dps* | DNA protection during starvation protein | SOPE01000100.1_00583 |
| *fixK* | Nitrogen fixation regulation protein FixK | SOPE01000100.1_00582 |
| *gerN_2* | Na(+)/H(+)-K(+) antiporter GerN | SOPE01000100.1_00572 |
| *gerN_3* | Na(+)/H(+)-K(+) antiporter GerN | SOPE01000100.1_00795 |
| *gltC_2* | HTH-type transcriptional regulator GltC | SOPE01000100.1_01177 |
| *rsmF* | Ribosomal RNA small subunit methyltransferase F | SOPE01000100.1_00066 |
|  | putative HTH-type transcriptional regulator | SOPE01000100.1_00164 |
| *iphP* | Tyrosine-protein phosphatase | SOPE01000100.1_00309 |
| *yhdG_1* | putative amino acid permease YhdG | SOPE01000100.1_00566 |
| *toxA_1* | Toxin A | SOPE01000100.1_00599 |
| *xerC_2* | Tyrosine recombinase XerC | SOPE01000100.1_00640 |
| *lexA_1* | LexA repressor | SOPE01000100.1_00645 |
|  | putative ABC transporter ATP-binding protein | SOPE01000100.1_00665 |
|  | putative membrane protein | SOPE01000100.1_00668 |
|  | 2-oxoglutaramate amidase | SOPE01000100.1_00771 |
| *Int-Tn_1* | Transposase from transposon Tn916 | SOPE01000100.1_00951 |
| *Int-Tn_2* | Transposase from transposon Tn916 | SOPE01000100.1_00963 |
|  | DegV domain-containing protein | SOPE01000100.1_01003 |
| *dacA_2* | D-alanyl-D-alanine carboxypeptidase DacA | SOPE01000100.1_01019 |
| *crcB_1* | Putative fluoride ion transporter CrcB | SOPE01000100.1_01078 |
| *toxA_3* | Toxin A | SOPE01000100.1_01117 |
|  | Nitronate monooxygenase | SOPE01000100.1_01244 |
| *smc_4* | Chromosome partition protein Smc | SOPE01000100.1_01299 |
| *immR_2* | HTH-type transcriptional regulator ImmR | SOPE01000100.1_01374 |
| *gspA* | General stress protein A | SOPE01000100.1_00356 |
| *hexR* | HTH-type transcriptional regulator HexR | SOPE01000100.1_01015 |
| *immR_1* | HTH-type transcriptional regulator ImmR | SOPE01000100.1_00643 |
| *ldhD* | D-lactate dehydrogenase | SOPE01000100.1_00143 |
| *licB* | Lichenan-specific phosphotransferase enzyme IIB component | SOPE01000100.1_01211 |
| *licT* | Transcription antiterminator LicT | SOPE01000100.1_00997 |
| *lyc* | Autolytic lysozyme | SOPE01000100.1_01122 |
| *mrp* | Iron-sulfur cluster carrier protein | SOPE01000100.1_00568 |
| *nanE* | Putative N-acetylmannosamine-6-phosphate 2-epimerase | SOPE01000100.1_01014 |
| *ndkA* | Nucleoside diphosphate kinase | SOPE01000100.1_01168 |
| *oppA* | Oligopeptide-binding protein OppA | SOPE01000100.1_00666 |
| *parA* | Chromosome partitioning protein ParA | SOPE01000100.1_00846 |
| *pepQ_2* | Xaa-Pro dipeptidase | SOPE01000100.1_00714 |
| *pflA* | Pyruvate formate-lyase-activating enzyme | SOPE01000100.1_00080 |
| *pflB* | Formate acetyltransferase | SOPE01000100.1_00079 |
| *prtP* | PII-type proteinase | SOPE01000100.1_00631 |
| *radD* | Putative DNA repair helicase RadD | SOPE01000100.1_01381 |
| *rhaR* | HTH-type transcriptional activator RhaR | SOPE01000100.1_01005 |
| *rluD_3* | Ribosomal large subunit pseudouridine synthase D | SOPE01000100.1_01326 |
| *rpe* | Ribulose-phosphate 3-epimerase | SOPE01000100.1_00084 |
| *rpiA* | Ribose-5-phosphate isomerase A | SOPE01000100.1_00085 |
| *rpsN2* | Alternate 30S ribosomal protein S14 | SOPE01000100.1_00682 |
| *sdhA* | L-serine dehydratase, alpha chain | SOPE01000100.1_00569 |
| *sdhB* | L-serine dehydratase, beta chain | SOPE01000100.1_00570 |
| *sglT* | Sodium/glucose cotransporter | SOPE01000100.1_00571 |
| *smc_3* | Chromosome partition protein Smc | SOPE01000100.1_00881 |
| *sufB_1* | FeS cluster assembly protein SufB | SOPE01000100.1_00661 |
| *sufB_2* | FeS cluster assembly protein SufB | SOPE01000100.1_00664 |
| *sufS* | Cysteine desulfurase SufS | SOPE01000100.1_00663 |
| *sufU* | Zinc-dependent sulfurtransferase SufU | SOPE01000100.1_00662 |
| *tetO* | Tetracycline resistance protein TetO | SOPE01000100.1_00683 |
| *tphA1I* | Terephthalate 1,2-dioxygenase, reductase component 1 | SOPE01000100.1_00585 |
| *tuaB* | Teichuronic acid biosynthesis protein TuaB | SOPE01000100.1_00360 |
| *ugd* | UDP-glucose 6-dehydrogenase | SOPE01000100.1_00676 |
| *urdA* | Urocanate reductase | SOPE01000100.1_01174 |
| *wfgD* | UDP-Glc:alpha-D-GlcNAc-diphosphoundecaprenol beta-1,3-glucosyltransferase WfgD | SOPE01000100.1_00690 |
| *xerS* | Tyrosine recombinase XerS | SOPE01000100.1_01171 |
| *xre* | HTH-type transcriptional regulator Xre | SOPE01000100.1_00644 |
| *ydiM_1* | Inner membrane transport protein YdiM | SOPE01000100.1_00122 |
| *ydiM_2* | Inner membrane transport protein YdiM | SOPE01000100.1_01178 |
| *yhdG_2* | putative amino acid permease YhdG | SOPE01000100.1_00567 |
| *yidK* | putative symporter YidK | SOPE01000100.1_01210 |
| *yjaB* | putative N-acetyltransferase YjaB | SOPE01000100.1_01372 |
| *ykfC* | Gamma-D-glutamyl-L-lysine endopeptidase | SOPE01000100.1_01126 |
