# Supplementary material for: Functional and Genomic Characterization of Ligilactobacillus salivarius TUCO-L2 Isolated From Lama glama Milk: A Promising Immunobiotic Strain to Combat Infections
Source: Front Microbiol. 2020 Dec 8;11:608752. doi: 10.3389/fmicb.2020.608752 (PMC7752859; doi:10.3389/fmicb.2020.608752)
Supplement: Supplementary Table 7 — BAGEL 4 and blastp analysis for the search of bacteriocins in the genome of Ligilactobacillus salivarius TUCO-L2. [file Table_7.docx]

**[NADP+] (EC 1.2.1.79);Ontology_term=KEGG_ENZYME:1.2.1.24,KEGG_ENZYME:1.2.1.79**

- SOPE01000171.1 FIG CDS 5820 5963 .ID=fig|6666666.666329.peg.979;Name=Acetyltransferase SOPE01000172.1 FIG CDS 32 1675 ID=fig|6666666.666329.peg.980;Name=Oligopeptide ABC transporter%2C substrate-binding protein OppA (TC 3.A.1.5.1)
- SOPE01000172.1 FIG CDS 1675 2574 ID=fig|6666666.666329.peg.981;Name=Proline iminopeptidase (EC 3.4.11.5);Ontology_term=KEGG_ENZYME:3.4.11.5
- SOPE01000173.1 FIG CDS 46 399 ID=fig|6666666.666329.peg.982;Name=**Bacteriocin immunity protein**
- SOPE01000173.1 FIG CDS 609 1328 ID=fig|6666666.666329.peg.983;Name=Aminopeptidase C (EC 3.4.22.40);Ontology_term=KEGG_ENZYME:3.4.22.40
- SOPE01000174.1 FIG CDS 3 149 . + 0 ID=fig|6666666.666329.peg.984;Name=Ribonucleotide reductase of class III (anaerobic)%2C activating protein (EC
- SOPE01000312.1 FIG CDS 538 768 . - 1 ID=fig|6666666.666329.peg.1476;Name=**Bacteriocin prepeptide or inducing factor for bacteriocin synthesis**
- SOPE01000312.1 FIG CDS 925 1098 . - 1 ID=fig|6666666.666329.peg.1477;Name=**Bacteriocin prepeptide or inducing factor for bacteriocin synthesis**
- SOPE01000386.1 FIG CDS 207 557 . - 0 ID=fig|6666666.666329.peg.1643;Name=**bacteriocin immunity protein**
- SOPE01000386.1 FIG CDS 207 557 . - 0 ID=fig|6666666.666329.peg.1643;Name=**bacteriocin immunity protein**
